# Analysis of Histones H3 and H4 Reveals Novel and Conserved Post-Translational Modifications in Sugarcane

**DOI:** 10.1371/journal.pone.0134586

**Published:** 2015-07-30

**Authors:** Izabel Moraes, Zuo-Fei Yuan, Shichong Liu, Glaucia Mendes Souza, Benjamin A. Garcia, J. Armando Casas-Mollano

**Affiliations:** 1 Departamento de Bioquímica, Instituto de Química, Universidade de São Paulo, São Paulo, Brazil; 2 Epigenetics Program, Department of Biochemistry and Biophysics, Perelman School of Medicine, University of Pennsylvania, Philadelphia, Pennsylvania, United States of America; National Taiwan University, TAIWAN

## Abstract

Histones are the main structural components of the nucleosome, hence targets of many regulatory proteins that mediate processes involving changes in chromatin. The functional outcome of many pathways is “written” in the histones in the form of post-translational modifications that determine the final gene expression readout. As a result, modifications, alone or in combination, are important determinants of chromatin states. Histone modifications are accomplished by the addition of different chemical groups such as methyl, acetyl and phosphate. Thus, identifying and characterizing these modifications and the proteins related to them is the initial step to understanding the mechanisms of gene regulation and in the future may even provide tools for breeding programs. Several studies over the past years have contributed to increase our knowledge of epigenetic gene regulation in model organisms like Arabidopsis, yet this field remains relatively unexplored in crops. In this study we identified and initially characterized histones H3 and H4 in the monocot crop sugarcane. We discovered a number of histone genes by searching the sugarcane ESTs database. The proteins encoded correspond to canonical histones, and their variants. We also purified bulk histones and used them to map post-translational modifications in the histones H3 and H4 using mass spectrometry. Several modifications conserved in other plants, and also novel modified residues, were identified. In particular, we report O-acetylation of serine, threonine and tyrosine, a recently identified modification conserved in several eukaryotes. Additionally, the sub-nuclear localization of some well-studied modifications (i.e., H3K4me3, H3K9me2, H3K27me3, H3K9ac, H3T3ph) is described and compared to other plant species. To our knowledge, this is the first report of histones H3 and H4 as well as their post-translational modifications in sugarcane, and will provide a starting point for the study of chromatin regulation in this crop.

## Introduction

The DNA of Eukaryotes is associated with proteins to form a highly dynamic complex called chromatin. The chromatin is composed of nucleosomes, which consist of an octamer of histone proteins. Within the nucleosome, ~146 base pairs of DNA are wrapped around two copies of histones H2A, H2B, H3 and H4. Nucleosomes are bound by the linker histone H1, to form the lowest level of chromatin condensation, the 10-nm fiber. In the next level of compaction, the 10-nm fiber coils, originating the 30-nm fiber. During interphase, the chromatin is present mostly in the form of 10-nm fiber, parts of 30-nm fiber and regions folded in looped domains (reviewed in [1]). Highly condensed chromatin domains are predominantly associated with transcriptionally inactive and gene poor sequences, and are referred to as heterochromatin. In contrast, euchromatin includes the less compacted domains associated with high transcriptional activity.

The regulation of chromatin states plays a key role in the control of gene expression. Extensive work has demonstrated that histones are involved in gene regulation (reviewed in [2, 3]). This regulation is accomplished by covalent modifications, which occur on selective amino acids and that can be added or removed by chromatin-modifying enzymes [4]. These post-translational modifications (PTMs) of histones occur preferentially in the N-terminal tails and include methylation of lysine and arginine; acetylation of lysine; and phosphorylation of serine, threonine and tyrosine.

A variety of histone variants that replace their canonical forms have been reported. For instance, the centromeric histone H3 (CENH3) replaces H3 in the centromeric chromatin. Another H3 variant, H3.3, differs from H3 in only 4 positions and, in contrast to canonical H3, its incorporation to the chromatin is replication-independent in metazoans [5, 6]. Some PTMs have been reported for histone variants. For instance, phosphorylation of CENH3 occurs in maize [7], budding yeast [8], and in humans [9, 10], where it plays a crucial role in kinetochore assembly and faithful chromosome segregation.

Methylation of histones is associated with both transcriptional repression and activation, where the indexing of a chromatin state will depend on the particular residue being methylated [11]. The key regulators of these two opposed chromatin states are members of the Polycomb Group (PcG) and Trithorax Group (TrxG). While PcG proteins methylate H3K27 (a mark associated with silencing), TrxG proteins trimethylate H3K4, a mark for active genes [12].

The enzymatic addition of a methyl group to a histone is catalyzed by a histone methyltransferase. In contrast to other modifications, one amino acid residue can carry more than one methyl group. Lysine can be mono, di or trimethylated, whereas arginine can be mono or dimethylated. In Arabidopsis, H3K4me1, H3K4me2, H3K4me3, H3K9me3 and H3K27me3 are marks for euchromatin. In contrast, H3K9me1, H3K9me2, H3K27me1 and H3K27me2 are associated with heterochromatin [13]. Nevertheless, these marks are not always conserved in plants. For instance, H3K27me2 was only found in the euchromatin in barley (*Hordeum vulgare*) nuclei, but the same modification is a mark for heterochromatin in *Vicia faba* [13]. Also, a previous study demonstrated that in species with smaller genomes H3K9me2 is restricted to the heterochromatin, while the same mark was dispersed over the nuclei of species with larger genome size [14]. In contrast, methyl H3K4 is distributed in the euchromatin in all plant species studied so far.

While methylation is associated to two opposed chromatin states, acetylation is usually involved in gene expression. The link of acetylation with transcriptional activation has been confirmed by Brownell and colleagues [15], who identified the first histone acetyltransferase (HAT) activity in Tetrahymena GCN5, a protein originally described as a gene activator. Histones are acetylated at lysine residues, a reaction catalyzed by HATs. The removal of acetyl groups lead to gene repression and is carried out by histone deacetylases (HDACs). The addition of an acetyl group to a lysine residue decreases its affinity for DNA, changing the nucleosome conformation, thus increasing the accessibility of regulatory proteins to the chromatin [16]. In plants, the lysines 9, 14, 18, 23, 27 and 56 of histone H3 and 5, 8, 12, 16 and 20 of histone H4 are acetylated [17–21].

O-acetylation of serine, threonine and tyrosine residues in the canonical histone H3 was only recently identified, though their function is still unknown [22]. In human cells, H3S10ac levels are increased during S phase and also in pluripotent cells. These observations suggest a role for this modification during DNA replication and in the maintenance of the pluripotent state and/or cell differentiation [22]. Britton et al. [22] proposed that during these processes H3S10ac may function as an antagonist to H3S10ph in a way similar to the blockage of MAP kinase phosphorylation by serine O-acetylation mediated by the YopJ acetyltransferase. However, this hypothesis remains to be experimentally demonstrated.

Histone phosphorylation is involved in a wide range of cell activities, such as mitosis, meiosis, cell death, replication, recombination and DNA repair [23]. Kinases are the proteins responsible for the addition of a phosphate group to the four core histones and also to the histone H1. Residues that can be phosphorylated are serine, threonine and tyrosine, although phosphorylation of tyrosine has been found only in mammals. Histone H3 phosphorylation is often associated with chromatin condensation during cell division. In plants, H3 phosphorylation of serine residues correlates with the pericentromeric region, where it may serve as recognition site for kinetochore assembly (reviewed in [24]). In contrast, phosphorylation of threonine is dispersed along the entire length of the chromosomes and seems to be involved in condensation and sister chromatid cohesion. During interphase, H3S10ph has been linked to activation of gene expression in mammals [25]. A correlation between transcriptional activation on interphase and histone H3 phosphorylation has only been demonstrated in plants as a response to external stimuli, indicating that H3S10ph might play a role in stress response [26].

Sugarcane (*Saccharum sp*.) is a tropical/subtropical grass of the Poaceae family, widely used for white sugar and bioethanol production. Brazil is responsible for more than half of total global sugar production. Because of its economic importance, research on sugarcane, especially with the goal to identify genes of interest for breeding, is on the rise. As a result, genome, transcriptome and proteome resources for sugarcane are becoming available. The SUCEST project, which focused on the study of the sugarcane transcriptome, since its beginning generated 237,954 ESTs, assembled in 43,141 clusters or sugarcane assembly sequences (SAS) that represent putative transcripts [27]. However, the study of epigenetic mechanisms in sugarcane remains completely unexplored. Taking into consideration that a complete understanding of gene regulation also requires knowledge of the proteins that mediate the accessibility to the DNA, the aim of the present study was to identify and initially characterize histone genes and their proteins in sugarcane. For that, we first identified candidate genes for histones H3 and H4. Next, we mapped PTMs, i.e. acetylation, methylation and phosphorylation, in canonical histones H3 and H4 and in their variants using mass spectrometry. Histone PTMs conserved in other organisms, as well as newly described PTMs were identified in both H3 and H4 histones, and the presence of the most well known modifications was confirmed by Western blotting. Additionally, the sub-nuclear localization of the same histone PTMs confirmed by Western blotting was investigated by immunostaining and compared to other plant species. To our knowledge, this is the first report of histones H3 and H4 as well as their PTMs in sugarcane and will provide a starting point to the study of chromatin regulation in this crop.

## Material and Methods

### Homology searches and phylogenetic analyses

Sugarcane histone H3 and H4 genes were identified by BLASTP and TBLASTN searches in the SUCEST sugarcane EST database (http://sucest-fun.org/wsapp). Arabidopsis histone H3.1 (HTR2, AT1G09200), histone H3.3 (HTR4, AT4G40030), histone H4.1 (HFO1, AT3G46320) and rice CENH3 (Os05g0489800) were used as queries in the BLAST searches. EST clusters, referred to as Sugarcane Assembled Sequences (SAS), showing high homology (cut off 1e-20) to either histone were considered for further analysis. CENH3 proteins in other species were identified by BLASTP searches in the GeneBank database (http://www.ncbi.nlm.nih.gov/genbank).

Sequences corresponding to CENH3 proteins were aligned using ClustalX [28] and the MEGA program v 6.0.5 [29] was used to obtain a neighbor-joining tree [30] with Poisson-corrected amino acid distances and bootstrap values based on 1000 pseudoreplicates.

### Plant growth, nuclei isolation and histone purification

Sugarcane (*Saccharum sp*.) cultivar SP80-3280 was propagated from stem cuttings and grown in a 12 h light/12 h dark photoperiod at 26°C for six months. Nuclei were isolated from sugarcane according to Bowler et al. [31] with small modifications. Briefly, 40 g of material was blended in the presence of 300 ml of cold Nuclei grinding buffer (1 M hexylene glycol, 10 mM PIPES/KOH pH 7.0, 10 mM MgCl2, 10 mM 2-mercaptoethanol, 0.5 mM PMSF, 10 mM NaF, 20 mM sodium butyrate), Triton X-100 was then added to 0.5%. After 10 min incubation, the nuclei were pelleted by centrifugation for 10 min at 1000 g, resuspended in Nuclei wash buffer (0.5 M hexylene glycol, 10 mM PIPES/KOH pH 7.0, 10 mM MgCl2, 0.2% Triton X-100, 10 mM 2-mercaptoethanol, 0.5 mM PMSF, 10 mM NaF, 20 mM sodium butyrate) and pelleted again by centrifugation for 10 min at 4000 g. Bulk histones were then extracted from the nuclear pellet using the cation exchange resin Biorex-70 (Bio-rad) as described by Waterborg et al. [32].

### NanoLC-MS/MS and data analysis

Total histones from sugarcane leaf rolls were subjected to chemical derivatization [33] using propionic anhydride (Sigma-Aldrich) and digested with sequencing grade trypsin at a 10:1 substrate to enzyme ratio for 6 hours at 37°C. The digested peptides were treated with an additional round of propionylation for the purpose of adding propionyl group to the newly generated N-terminus. Peptides were desalted using C18 extracted mini disk (Empore 3M, MN, USA) and dissolved in 0.1% formic acid. Samples were loaded via an autosampler (EASY-nLC, Thermo Fisher Scientific Inc) onto a homemade 75 μm reversed phase analytical column packed with 15 cm C18-AQ resin (3μm particle sizes, 120 Å pore size) at a rate of 550 nL/min. Peptides were chromatographically resolved on a 71-min 2–98% solvent B gradient (solvent A = 0.1% formic acid, solvent B = 100% acetonitrile) at a flow rate of 250 nL/min. The eluted peptides were electrosprayed through a PicoTip emitter (New Objective Inc, Woburn, MA) into and detected by LTQ Orbitrap Velos mass spectrometer (Thermo Fisher Scientific Inc) with a resolution of 60,000 for full MS spectrum followed by MS/MS spectra obtained in the ion trap.

After the MS data were generated, a database searching workflow was used to identify histone modifications [34]. The sugarcane database was searched, including H2A, H2B, H3, and H4 (54 entries in total). Search parameters were set for the database search engine pFind 3.0 [35, 36], including precursor mass tolerance ±10 ppm, fragment mass tolerance ±0.4 Da, trypsin cleaving after arginine and up to 2 miscleavages, peptide N-terminal propionylation (propionyl[Peptide N-term]/+56.026) as the fixed modification. To obtain more identification for different kinds of peptides, we searched the following variable modifications: unmodified peptides (un) with propionyl[K]/+56.026, acetylated peptides (ac) with acetyl[KSTY]/+42.011, mono-methylated peptides (me1) with methyl+propionyl[K]/+70.042 and methyl[R]/+14.015, di-methylated peptides (me2) with dimethyl[KR]/+28.031, tri-methylated peptides (me3) with trimethyl[K]/+42.047, phosphorylated peptides (ph) with phospho[STY]/+79.966, and oxidation[M]/15.9949. The target-decoy approach was used to filter the search results, in which the false discovery rate was less than 1% at the peptide level. The filtered MS/MS spectra were also checked using the EpiProfile program [37]. In the case of trimethylation, which differs from acetylation by only 0.03639 Da, the assignment of either modification to a given peptide was done by high accuracy mass measurement. However, chromatographic retention time (trimethylated peptides elute earlier than their acetylated isoforms) was also used to verify the correct assignment of trimethyl and acetyl groups.

The abundance of each peptide isoform was determined by peak integration of the ion chromatogram with the EpiProfile program [37]. Relative amount of each peptide isoform was then calculated as the area under the curve for each peak and expressed as the percentage of the total peptides (the sum of all isoforms of a particular peptide).

### Nuclei preparation and immunostaining

Nodes of sugarcane plants were kept at 35°C in a humidity chamber to induce adventitious root growth. Roots were harvested and fixed for 20 min with ice-cold 4% (w/v) paraformaldehyde in MTSB buffer (50 mM PIPES, 5 mM MgSO4, 5 mM EGTA, pH 6.9). Root tips were cut and digested for 70 min at 37°C with an enzyme mixture (10% macerozyme, 5% pectinase, 5% cellulase Onozuka R-10 in MTSB) and squashed in a drop of MTSB buffer. The following antibodies were used diluted in a AD buffer (1% BSA in MTSB): anti-H3K9me2 (1:200, 07–212, Millipore), anti-H3K4me1 (1:200, 07–436, Millipore), H3K4me3 (1:200, 07–473, Millipore), H3K27me3 (1:200, 07–449, Millipore), H3K9ac (1:100, ab4441, Abcam), H3T3ph (05-746R; Millipore) and RNA Polymerase II (1:300, 05–623, Upstate). Slides were incubated with the antibody solution overnight. After washing in MTSB, immunofluorescence signals were detected with Anti-Rabbit IgG-Rhodamine (1:300; # 31685, Pierce). For documentation, slides were analyzed in a Nikon Eclipse TE300 microscope and images registered using a Nikon DS-Ri1 camera.

### Immunoblot assays

The in vivo status of specific modifications was examined on histones purified from leaves and stems. Approximately 5 μg of histones were separated by SDS-PAGE, electroblotted onto nitrocellulose membranes and probed with modification specific antibodies. Methylation states on the histone H3 were determined using antibodies against H3K4me1, H3K4me3, H3K9me2 or H3K27me3. H3 acetylation status was examined using an antibody against acetyl H3K9 whereas H3 phosphorylation was determined with an antibody against phosphorylated H3T3. Catalog numbers of the antibodies are the same as for immunostaining. Sample loading was adjusted relative to the signal obtained with an anti-H3 antibody (07–690; Millipore) that recognizes its C-terminal region.

## Results

In order to identify expressed genes encoding homologs of the histones H3 and H4, we searched the sugarcane EST database (http://sucest-fun.org/wsapp/). Blast with H3 and H4 proteins from Arabidopsis and rice as queries, revealed multiple EST clusters or Sugarcane Assembled Sequences (SAS) representing transcripts corresponding to these histones (S1 Table). Due to the high polyploidy and the heterozygous nature of the sugarcane genome it is unknown if the SAS identified correspond to different loci or to multiple alleles in the same locus.

### Sugarcane histone H3 genes

40 SAS with homology to histone H3 from Arabidopsis and other organisms were found in the Sugarcane EST collection (S1 Table). The proteins encoded by the sugarcane SASs are highly conserved with the histone H3 from Arabidopsis, rice and maize (S1 Fig). Based on the presence of four amino acid replacements [17], 17 SASs were found to encode the canonical H3 (H3.1) and 18 of them the replacement histone variant H3.3. All the proteins encoded by the SAS classified as H3.1 showed 100% homology between them and were collectively named Ss_H3.1 following the recommendations from Talbert et al. [38]. Similarly, all proteins from SASs classified as H3.3 were identical and named Ss_H3.3. Alignment of Ss_H3.1 and Ss_H3.3 indicates that they share 100% homology with H3.1 and H3.3 from Arabidopsis, rice and maize (S1 Fig). Two SAS were also found to encode CENH3 isoforms similar to those previously described by Nagaki and Murata [39]. The two isoforms identified in this study, Ss_CENH3a-b, are nearly identical and differ in only three amino acid residues (Figure A in S2 Fig). Phylogenetic analysis between CENH3 from *Saccharum sp*. and proteins from other monocots indicates that the two isoforms present in sugarcane likely evolved after their divergence from sorghum. CENH3 isoforms from *Luzula nivea* and *Hordeum vulgare* are also more similar to each other than to proteins from other species suggesting that CENH3 isoforms likely arose multiple times during monocot evolution (Figure B in S2 Fig). The three remaining SAS with homology to H3 encoded truncated proteins with similarity to either H3.1 or H3.3, but with additional amino acid changes. However, since these SAS are represented by single EST reads it was difficult to determine if they truly correspond to histone variants and/or the result of sequencing errors.

### Histone H4 genes

In contrast to H3, in which several variants fulfilling different roles are present, H4 function has remained invariable throughout eukaryotic evolution [40]. This observation appears to apply to Arabidopsis in which all the H4 genes encode for identical proteins and, to date, H4 variants remain unknown. In sugarcane we identified 24 SAS with homology to histone H4 (S2 Table). From these, 21 SAS encode proteins with 100% homology to the canonical H4 from Arabidopsis, rice and maize and were classified as Ss_H4.1 (S2 Table, S3 Fig). Another SAS encoding an H4 with a single nucleotide substitution (Y72C) was also found in the sugarcane genome and was named Sc_H4.2. The identification of an H4 variant in sugarcane is supported by multiple EST reads for the SAS encoding this protein. Another two H4 variants highly similar to Ss_H4.1 were also identified but represented only by single reads encoding truncated proteins. Thus, the same constraints operating in other eukaryotes may apply to H4 sugarcane in which only one H4 variant appears to have evolved.

### Sugarcane histone purification and analysis

To further characterize the histones from sugarcane, we decided to catalog the modifications present in histones H3 and H4. We started by purifying total histones from leaf roll nuclei using acid extraction and cation exchange chromatography. The protein profile of the isolated histones after polyacrylamide gel electrophoresis (SDS-PAGE) was very similar to that of cauliflower [31] and alfalfa [41] and indicates that they are mostly free of non-histone proteins (S4 Fig). The isolated bulk histones were then analyzed by nanoLC-MS/MS and the acquired data was searched against databases including the predicted sugarcane histones H3 and H4 and proteins from the sugarcane SAS collection. This “one-pot” approach was shown capable of detecting the same post-translational modifications on histones H3 and H4 as with the less advantageous (and more time consuming) off-line HPLC or SDS-PAGE purification of individual histones [42].

### Post-translational modifications in histone H3

**Fig 1:**
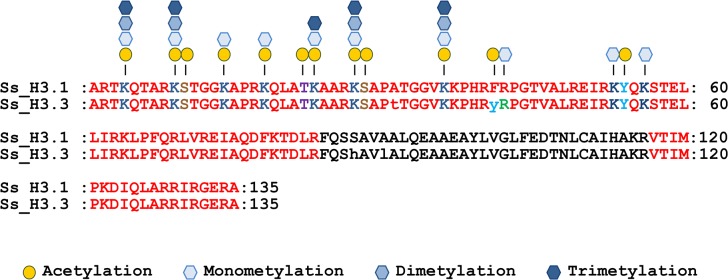
Post-translational modifications identified in sugarcane histone Ss-H3.1 and Ss_H3.3 variant. Amino acid residues covered by the peptides identified by the MS/MS analysis are indicated in red. The modification sites identified are shown on top of the sequence and the amino acid residues highlighted in blue (lysine), green (arginine), brown (serine), purple (threonine) and light blue (tyrosine). The first amino acid methionine was omitted from the sequence.

NanoLC-MS/MS analysis of sugarcane bulk histones yielded several peptides corresponding to sugarcane histones Ss_H3.1 and Ss_H3.3 (S3 Table). Total peptide coverage for H3.1 and H3.3 was 75% and included the complete histone tail and part of the core domain including the C-terminal portion of the protein (Fig 1). Due to the similarity between H3.1 and H3.3, several peptides could not be assigned to a particular H3 type. However, some peptides specific to each variant were identified confirming that both proteins are present in the isolated histones.

**Fig 2:**
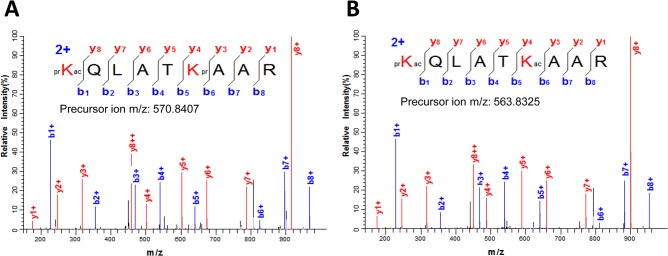
Lysine acetylation in sugarcane histone H3. (A) MS/MS spectrum of the doubly-charged ion at *m*/*z* 570.8407 corresponding to the H3 peptide prKacQLATKprAAR (residues 18–26) where K18 is acetylated. (B) Fragment ions of the recorded in MS/MS spectrum for the [M+2H]^2+^ ion (*m*/*z* 563.8325) matches to the peptide prKacQLATKacAAR acetylated at positions K18 and K23. Sequence of the modified peptide and the measured mass of the precursor ion are shown in the figure inset. N-terminal and lysine propionylation, products of the chemical derivatization, are indicated by pr.

The detected post-translational modifications, including acetylation and methylation, mapped to their respective amino acid positions are shown in Fig 1. Furthermore, S3 Table summarizes all the modified peptides sequenced by MS/MS from Ss_H3.1 and Ss_H3.3. In Ss_H3.1 and Ss_H3.3, we found acetylation at lysines 4, 9, 14, 18, 23, 27 and 36. As an example of identification of acetylation in a particular site, Fig 2A shows the MS/MS spectrum of the doubly-charged ion at *m*/*z* 570.8407 for a peptide generated in a trypsin digestion of the bulk histones. The observed mass corresponds to a peptide with the sequence KQLATKAAR (residues 18–26 from either histone H3.1 or H3.3) containing two propionyl and one acetyl groups. The observed fragmentation ions, “b” and “y” in this spectrum match the peptide prKacQLATKprAAR where K18 is acetylated and, the N-terminus and lysine 23 are propionylated (Fig 2A). Propionylation of the unmodified lysine 23 and the N-terminus is a product of the chemical derivatization of histones before nanoLC-MS/MS analysis. Not only single acetylated peptides, but also peptides with two acetyl groups were identified (S3 Table). In the case of the peptide prKacQLATKacAAR, fragment ions of the recorded MS/MS spectrum for the [M+2H]^2+^ ion (*m*/*z* 563.8325) match to this peptide acetylated at position K18 and K23 (Fig 2B).

**Fig 3:**
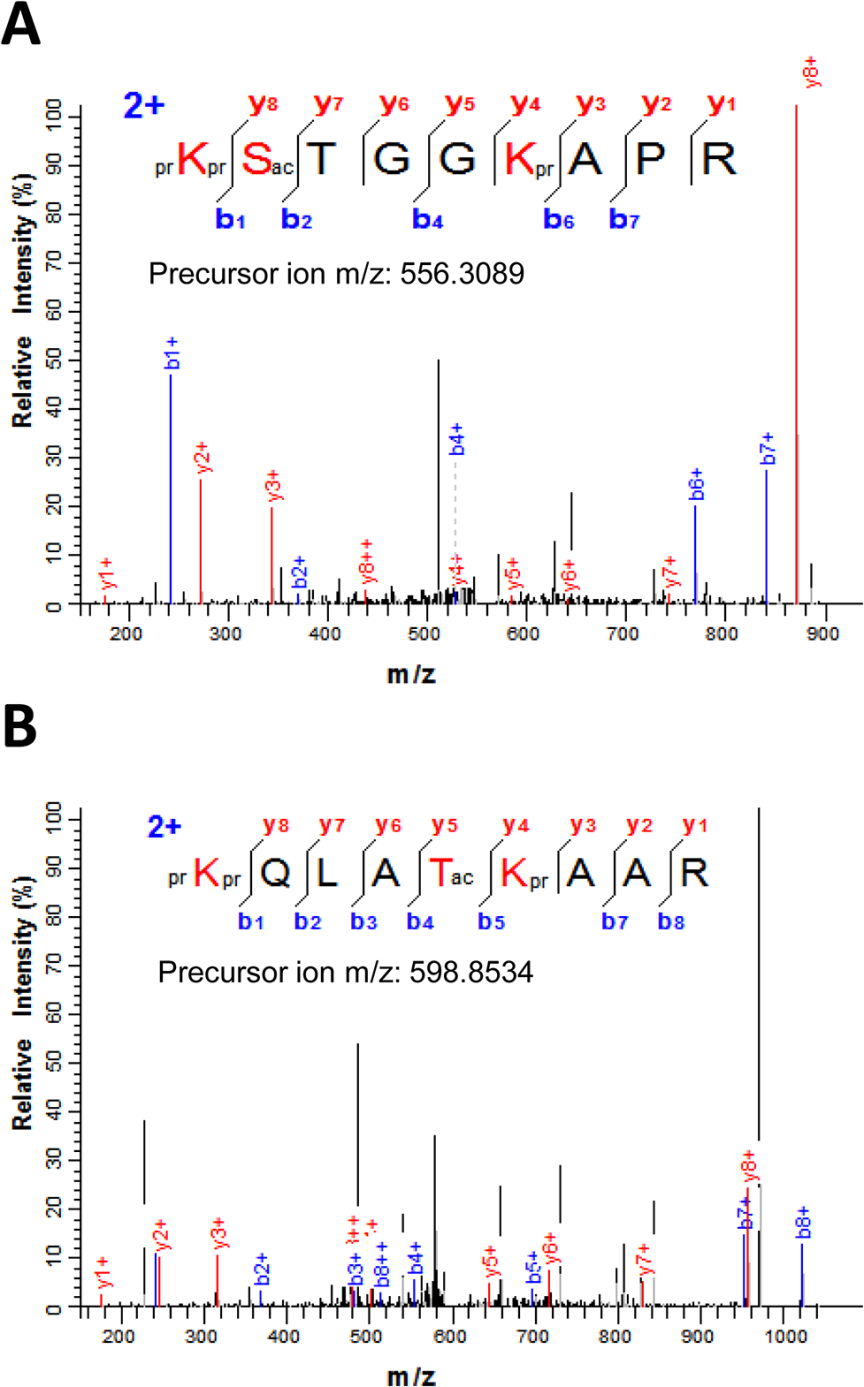
Serine/threonine O-acetylation in sugarcane histone H3. (A) MS/MS spectrum of the [M+2H]^2+^ ion (*m*/*z* 556.3089) that matched the histone H3 peptide prKSacTGGKprAPR (residues 9–17) where S10 is acetylated. (B) MS/MS spectra of the doubly-charged precursor ion at *m*/*z* 598.8534 corresponding to H3T22 acetylation in the H3 peptide prKQLATacKprAAR (residues 18–26). Sequence of the modified peptide and the measured mass of the precursor ion are shown in the figure inset. N-terminal and lysine propionylation, products of the chemical derivatization, are indicated by pr.

Recently, low level O-acetylation at serine, threonine and tyrosine (S/T/Y) was shown to occur in the histone H3 of several model organisms [22]. H3S10ac in particular appears to be conserved from humans to yeast [22]. In our nanoLC-MS/MS analysis of sugarcane histones, we identified several peptides acetylated at H3S10, H3T22, H3S28, H3Y41 and H3Y54 (S3 Table). Acetylation of these residues, with exception of H3Y41, has been shown to occur in other organisms [22]. H3S10ac was identified from the MS/MS spectrum of the [M+2H]^2+^ ion (*m*/*z* 556.3089) that matched the peptide prKSacTGGKAPR (residues 9–17) where H3S10 is acetylated (Fig 3A). However, the presence of multiple unassigned peaks of high intensity in the middle of the MS/MS spectrum in which H3S10ac was identified indicate there is a mix of peptides. Some of these peaks can be match to y5, y6 and y7 ions corresponding to prKme1SacTGGKacAPR (S5 Fig). Therefore, this spectrum corresponds to a mixture of H3S10ac and H3K9me1S10acK14ac peptides.

H3T22 acetylation in the peptide prKQLATacKprAAR (residues 18–26) was established from fragmentation ions of the MS/MS spectra of the doubly-charged precursor ion at *m*/*z* 598.8534 (Fig 3B). However, there are about ten unassigned peaks of high intensity, which indicates that there is at least another peptide in the spectrum. Eight out the ten high abundant peaks in this spectrum are matched to the peptide prKacQLATacKme1AAR (S6 Fig). These observations suggest that this spectrum corresponds to a mix of peptides containing H3T22ac and H3K18acT22acK23me1.

Double modified peptides containing O-acetyl S/T/Y were also detected almost exclusively associated to lysine or arginine methylation. For instance, we found H3S10ac together with H3K9me1, H3K9me3 and H3K14me1; H3T22ac with H3K23me1 and H3K18ac; H3S28ac with H3K27me1, H3K27me3 and H3K36me1; H3Y41ac with H3R42me1; and H3Y54ac with H3K56me1 (S3 Table).

**Fig 4:**
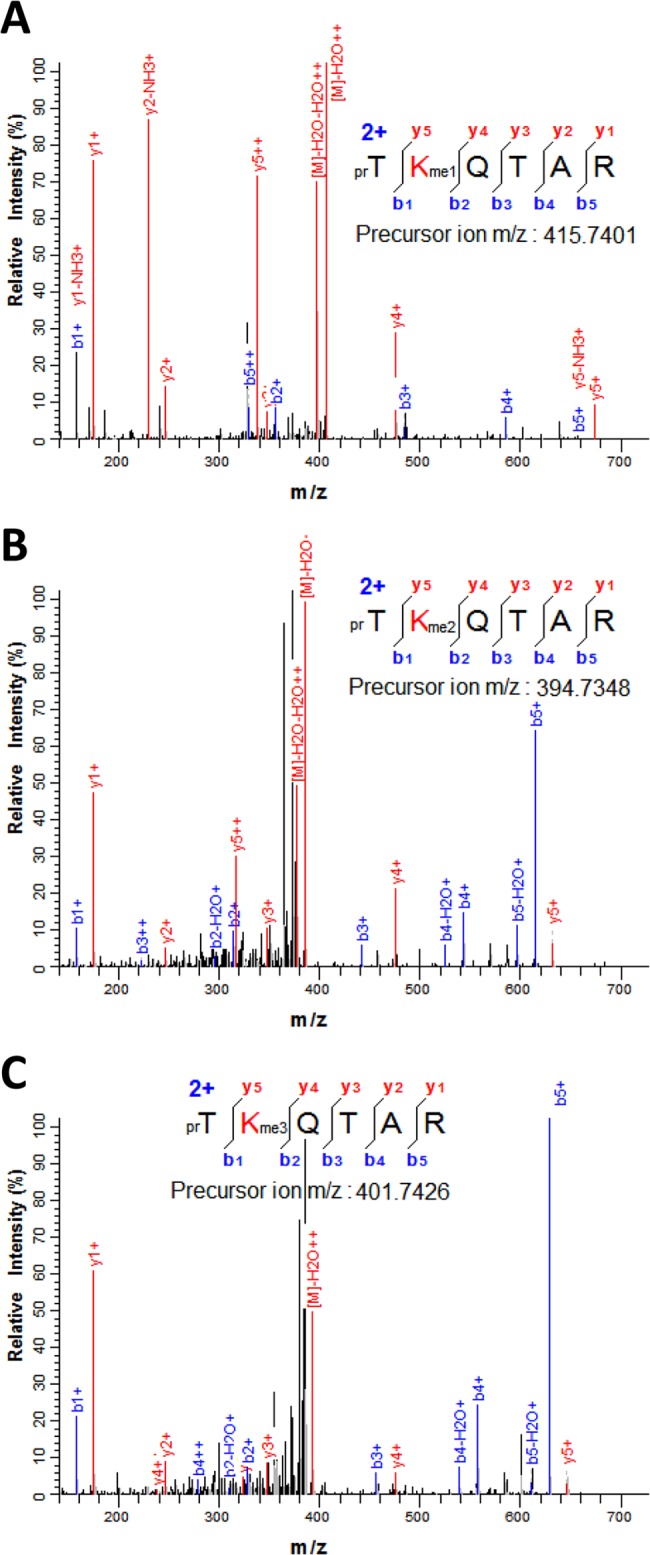
Detection of H3K4 methylation in sugarcane. (A) MS/MS fragmentation pattern recorded on the [M+2H]^2+^ ion at *m*/*z* 415.7401 that matches the histone H3 peptide (residues 3–8) prTKme1QTAR containing monomethyl K4. (B) MS/MS spectrum of the doubly-charged ion at *m*/*z* 394.7348 corresponding to the H3 peptide prTKme2QTAR. (C) MS/MS spectrum recorded on the [M+2H]^2+^ ion (*m*/*z* 401.7426) that corresponds to the peptide prTKme3QTAR. Sequence of the peptide and the measured mass of the precursor ion are shown in the figure inset. N-terminal propionylation, product of the chemical derivatization, is indicated by pr.

Lysine and arginine methylation were also detected in several positions including the globular domain of histones H3.1 and H3.3 variant (Fig 1A). Methylation of lysines 4, 9, 14, 18, 23, 27 and 36 were found in the N-terminal tail whereas lysine 53 and 56 and arginine 42 could be detected in the globular domain. In the case of H3K4 methylation, the peptide prTKme1QTAR (H3 residues 3–8) monomethylated at H3K4 was identified from the MS/MS fragmentation pattern of the [M+2H]^2+^ ion at *m*/*z* 415.7401 (Fig 4A). Two other MS/MS spectra from the doubly-charged ions at *m*/*z* 394.7348 and *m*/*z* 401.7426 were also found to correspond to the peptide TKQTAR where K4 is di- and trimethylated, respectively (Fig 4B and 4C).

We could not reliably assign any serine and threonine phosphorylation to the histone H3 from our NanoLC-MS/MS analysis. Histone H3 phosphorylation in plants has been reported for several residues, i.e. H3T3, H3T6, H3S10, H3S28, H3T22, mainly by the use of modification specific antibodies. The levels of these PTMs are usually very low and restricted to few genomic regions during interphase (reviewed in [24]). In contrast, three independent mass spectrometry analysis of histone H3 from Arabidopsis and soybean failed to detect S/T phosphorylation in these sites, even after phosphopeptide enrichment [17, 18, 20]. The inability of the MS analysis to detect phosphorylation may be attributed to the low levels of this modification present in interphase cells but also to the hydrolysis of phosphorylation during sample preparation. Indeed, two putative phosphorylated peptides corresponding to histone H3 were detected based on accurate mass measurement and retention time. However, due to their low abundance the mass spectrometer did not obtain MS/MS spectra for them and a confident assignment of the phosphorylated residues could not be carried out.

**Fig 5:**
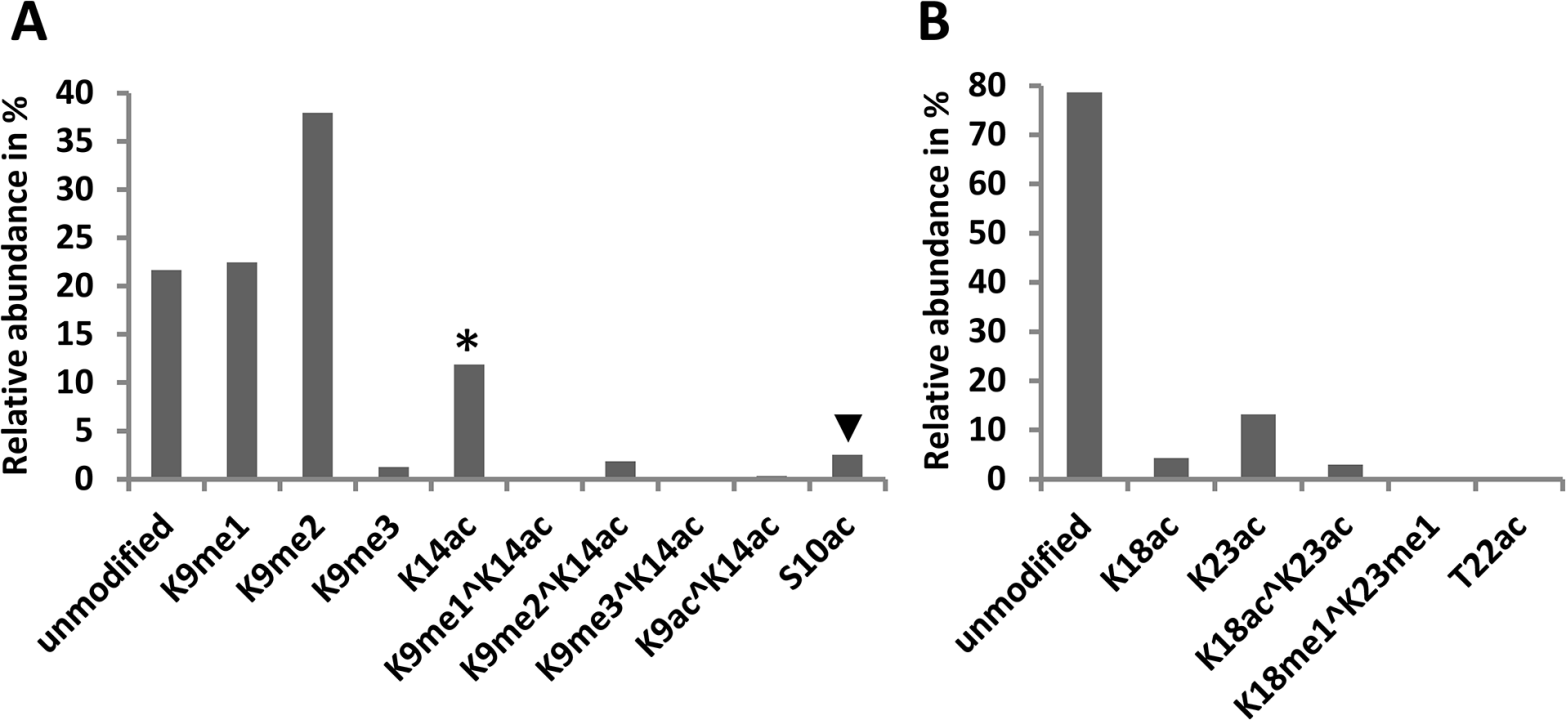
Relative abundance of histone H3 (residues 9–26) acetylation and methylation in sugarcane. (A) Percent relative amounts of peptide isoforms containing residues 9–17 of histone H3. * Peptide isoforms containing a single acetylation at K9 or K14 could not be separated by nanoLC. ▼ Peptide isoforms containing a single acetylation on S10 or T11 could not be separated by nanoLC. (B) Relative amounts of peptide isoforms containing residues 18–26 of histone H3. Only the most abundant isoforms are shown.

Propionylation of histones blocks lysine residues allowing trypsin to cleave only C-terminal to arginine and generating defined peptides. We took advantage of this property and used the ion currents of each peptide isoforms to calculate the relative abundance of their associated modifications [17]. In the histone H3 tail we calculated the relative abundance of modifications present in the peptides corresponding to residues 9–17, 18–26 and 27–40. The predominant modifications on the H3 peptide at residues 9–17 was H3K9 methylation. The highest levels correspond to K9me1 (22%) and K9me2 (38%) whereas K9me3 was lower than 2% (Fig 5A). The levels of acetylation were lower than those observed in Arabidopsis, but still more than 10% of the lysines are acetylated at either K9 or K14 (Fig 5A). Since K9 and K14 acetylated peptides co-elute in the nanoLC, we used the MS/MS spectra to calculate the abundance of each modification. We found the levels of K9 acetylation to be 50 times lower than those of K14. Similarly, the levels of the doubly acetylated peptide (at K9 and K14) turned out to be very low compared to K14 acetylation and only 2% of the peptides contain the double modification, methyl K9 and acetyl K14 (Fig 5A). These observations indicate that in sugarcane K14 is the predominant acetylation mark and not K9. Peptides corresponding to residues 18–26 of histone H3 were found mostly unmodified (78%) or acetylated at K18 (4%), at K23 (13%) or at both residues (3%) (Fig 5B). Methylation of K18 and K23 on the contrary were shown to be present at very low levels (~0.1%) (Fig 5B). The relative abundance of serine and threonine (S/T) O-acetylation was very low (1% or less) in all the organisms examined which is in stark contrast to the much higher levels of lysine acetylation [22]. In sugarcane, we observed a similar disparity between the levels of S/T and lysine acetylation (Fig 5A and 5B). The relative level of H3T22ac is less than 0.5% whereas H3S10ac was at 2.5%. Although sugarcane H3S10ac, combined with H3T11ac, level is slightly higher than the 1% observed for other organisms, it is still very low compared to the relative levels of lysine acetylation.

**Fig 6:**
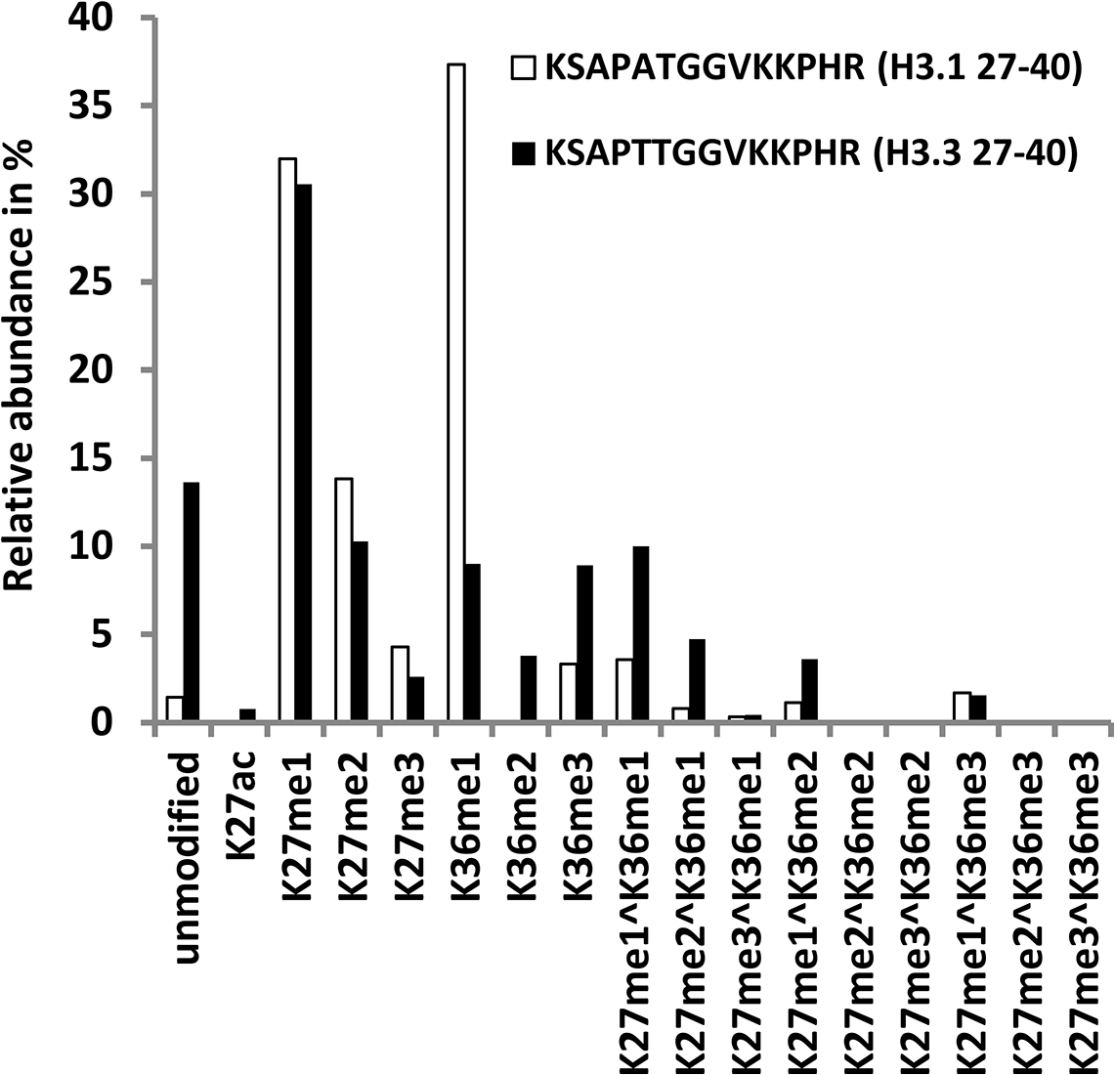
Comparative analysis of post-translational modifications in the canonical Ss_H3.1 and Ss_H3.3 variant. Relative amounts of the different modifications were calculated for the peptides corresponding to residues 27–40 of H3.1 (KSAPATGGVKKPHR) and H3.3 (KSAPTTGGVKKPHR). Only the most abundant isoforms are shown.

We identified an H3.3 variant in sugarcane that, like sequences of all the plant H3.3 identified so far, was different from the H3.1 in only four positions. Due to this sequence similarity, we could not differentiate between both variants for most of the length of the proteins, except for two peptides corresponding to residues 27–40 and 41–49 [17]. In this region we found acetylation and methylation at lysine K27 and K36. Relative quantification of the H3.1 peptides indicates that while K27 is highly methylated at this position (32% me1, 14% me2 and 4% me3), the levels of K27ac were extremely low (0.01%) (Fig 6). The position K36 was highly monomethylated (37%) whereas the di- and trimethyl forms were extremely low, > 0.01% and 3% respectively. Fragments methylated at both positions, i.e. 3% K27me1^K36me1, 0.8% K27me2^K36me1, 1% K27me1^K36me2 (Fig 6), were also detected. In the peptides corresponding to H3.3, the methylation levels of K27 were consistently lower than those for H3.1 (30% me1, 10% me2 and 2% me3) especially K27me3 (Fig 6). Acetylation of K27 was also found close to 1%. Compared to H3.1, the levels of H3K36me1 were lower in H3.3 (9%), yet fairly high levels of di- and trimethylation of H3K36 were found in this variant (respectively, 4% and 9%). The levels of the peptides modified at both K27 and K36 were also higher in H3.3, i.e. 10% K27me1^K36me1, 4.7% K27me2^K36me1, 3% K27me1^K36me2 (Fig 6).

In contrast to H3.1 and the variant H3.3, few peptides, covering 29.5% and 34.6% of Ss_CENH3.a and Ss_CENH3.b respectively, were found to correspond to sugarcane CENH3 (S7 Fig). We found in *Saccharum sp* cultivar SP80-3280 ESTs clusters encoding two isoforms of CENH3 (a and b). Interestingly, the nanoLC-MS/MS analysis identified peptides corresponding to both isoforms, indicating that they coexist in sugarcane.

### Post-translational modifications in histone H4

**Fig 7:**
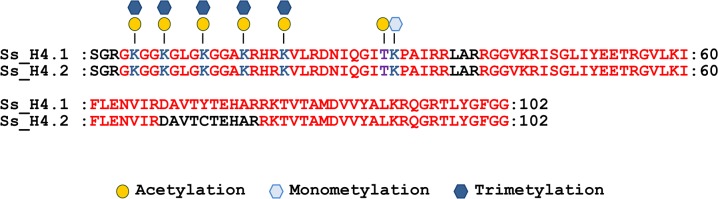
Post-translational modifications identified in the histone H4 from sugarcane. Amino acid residues identified by the nanoLC-MS/MS analysis are indicated in red. The modification sites identified are shown on top of the sequence and the amino acid residue highlighted in blue (lysine) and purple (threonine). The first amino acid methionine was omitted from the sequence.

In sugarcane we identified one protein with similarity to the canonical histone H4, Ss_H4.1, and a single variant, Ss_H4.2, with a single amino acid substitution Y72C. NanoLC-MS/MS analysis of bulk histone sequenced several peptides corresponding to histone H4. The protein coverage was 93.2% for Ss_H4.1 and 83.5% for the Ss_H4.2 variant (Fig 7). However, unique peptides generated from trypsin digestion of Ss_H4.2 were not found indicating that the variant may be present at very low levels, below the detection limit of the assay, or may be a tissue specific variant not expressed in leaf rolls. Therefore we consider all the modifications identified belonging only to Ss_H4.1.

**Fig 8:**
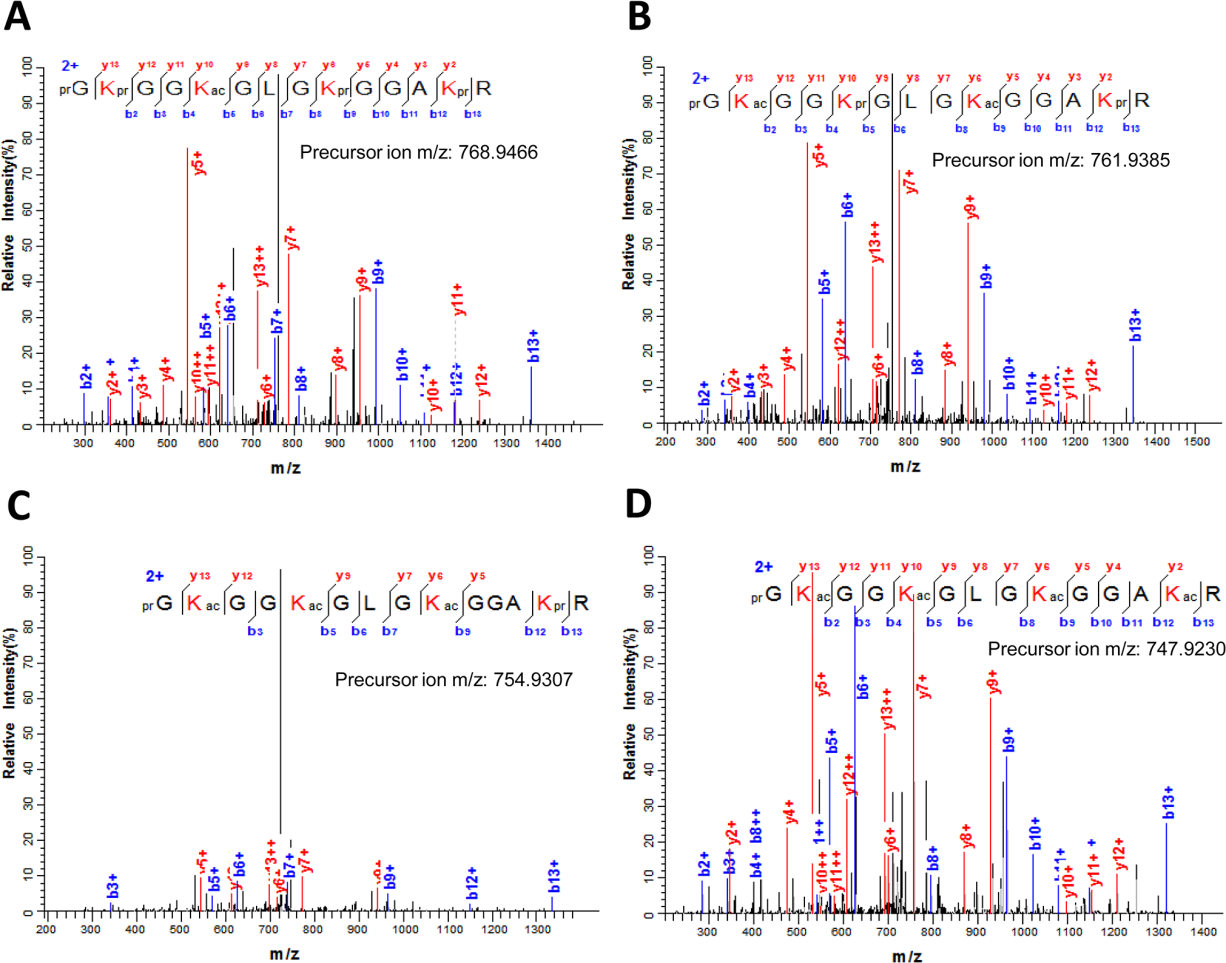
Lysine acetylation in sugarcane histone H4. (A) MS/MS spectrum of the doubly charged ion at *m*/*z* 768.9466 that corresponds to the histone H4 peptide (residues 4–17) prGKprGGKacGLGKprGGAKprR containing acetyl K8. (B) MS/MS spectrum of the [M+2H]^2+^ ion at *m*/*z* 761.9385 matching the H4 peptide prGKacGGKprGLGKacGGAKprR where K5 and K12 are acetylated. (C) MS/MS spectrum recorded on the [M+2H]^2+^ ion (*m*/*z* 754.9307) that corresponds to the peptide prGKacGGKacGLGKacGGAKprR where K5, K8 and K12 are acetylated. (D) The full acetylated peptide prGKacGGKacGLGKacGGAKacR at K5, K8, K12 and K16 was deduced from the MS/MS spectrum of the ion at *m*/*z* 747.9230. Sequence of the modified peptide and the measured mass of the precursor ion are shown in the figure inset. N-terminal and lysine propionylation, products of the chemical derivatization, are indicated by pr.

A list of all modified peptides sequenced in the nanoLC-MS/MS analysis corresponding to histone Ss_H4.1 is shown in S4 Table. The modifications detected on these peptides include acetylation and methylation (Fig 7). From the modifications detected on histone Ss_H4.1, acetylation was found in all lysines present in the histone tail-domain (residues 5–20) and in a single threonine at position 30. Lysine acetylation is not only present in a single site but peptides in which two, three and even four lysines are acetylated could also be identified. In the peptide GKGGKGLGKGGAKR (residues 4–17) containing four out of the five lysines acetylated, we could detect combinations of two (i.e. K5-K12, K8-K16) and three (i.e. K5-K8-K12, K8-K12-K16) lysines and the fully acetylated peptides (S4 Table). Examples of the peptides sequenced containing one to four acetylated lysines are given in Fig 8A single residue H4K8 in the peptide prGKprGGKacGLGKprGGAKprR (residues 4–17) was determined to be acetylated from the MS/MS spectrum recorded from the doubly-charged ion at *m*/*z* 768.9466 (Fig 8A). Meanwhile, the MS/MS spectrum of the doubly-charged ion (*m*/*z* 761.9385) was shown to correspond to the peptide prGKacGGKprGLGKacGGAKprR where both K5 and K12 are acetylated (Fig 8B). The same peptide corresponding to residues 4–17 but containing three (K5ac, K8ac, K12ac) and four (K5ac, K8ac, K12ac, K16ac) acetyl groups were deduced from the MS/MS spectra recorded for the ions at *m*/*z* 754.9307 and *m*/*z* 747.9230, respectively (Fig 8C and 8D).

**Fig 9:**
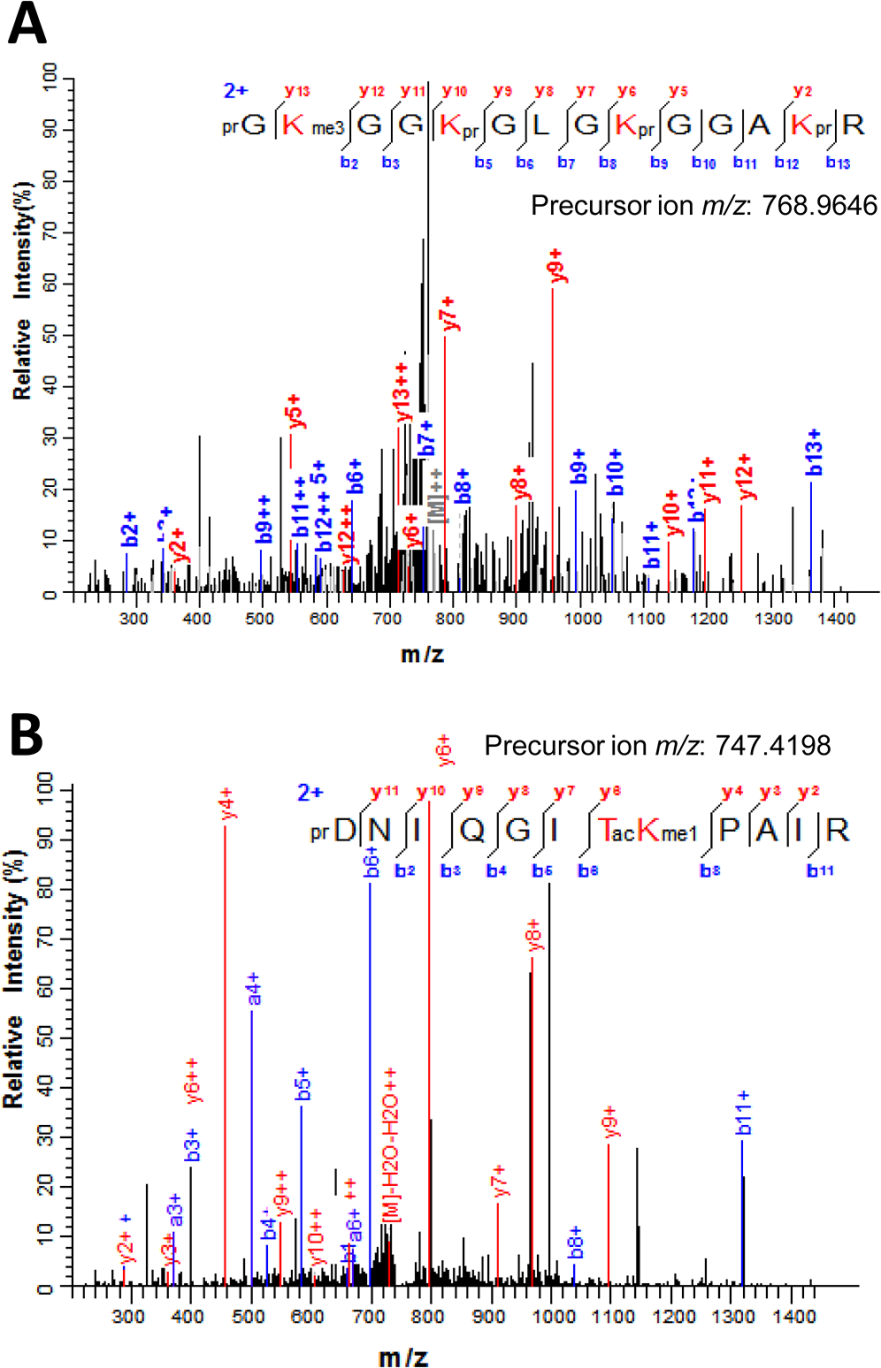
Lysine methylation and threonine acetylation in sugarcane histone H4. (A) MS/MS spectrum of the doubly charged ion at *m*/*z* 768.9646 indicating H4K5me3 in the H4 peptide prGKme3GGKprGLGKprGGAKprR (residues 4–17). (B) MS/MS spectrum [M+2H]^2+^ ion at *m*/*z* 747.4198 corresponding to the peptide prDNIQGITacKme1PAIR (residues 24–35) containing H4T30ac and H4K31me1. Sequence of the modified peptide and the measured mass of the precursor ion are shown in the figure inset. N-terminal and lysine propionylation, products of the chemical derivatization, are indicated by pr.

In the same way as acetylation, methylation was detected in all lysines corresponding to the tail domain in the histone H4 (Fig 7). For instance, H4K5me3 in the peptide prGKme3GGKprGLGKprGGAKprR (residues 4–17) was deduced from the MS/MS spectrum of the doubly-charged ion at *m*/*z* 768.9646 (Fig 9A). In addition to modifications in the tail domain, a lysine present in the globular domain, H4K31, was also found methylated. Methylation of H4K31 was identified from the MS/MS spectrum recorded from the [M+2H]^2+^ ion at *m*/*z* 747.4198 that matched the H4 peptide prDNIQGITacKme1PAIR (residues 24–35) were H4K31 is monomethylated (Fig 9B). H4T30 was also found acetylated in this same peptide. Thus, in contrast to lysine acetylation, methylation occurs not only in the histone tail, but also in the globular domain.

### Nuclear distribution of PTMs in sugarcane

**Fig 10:**
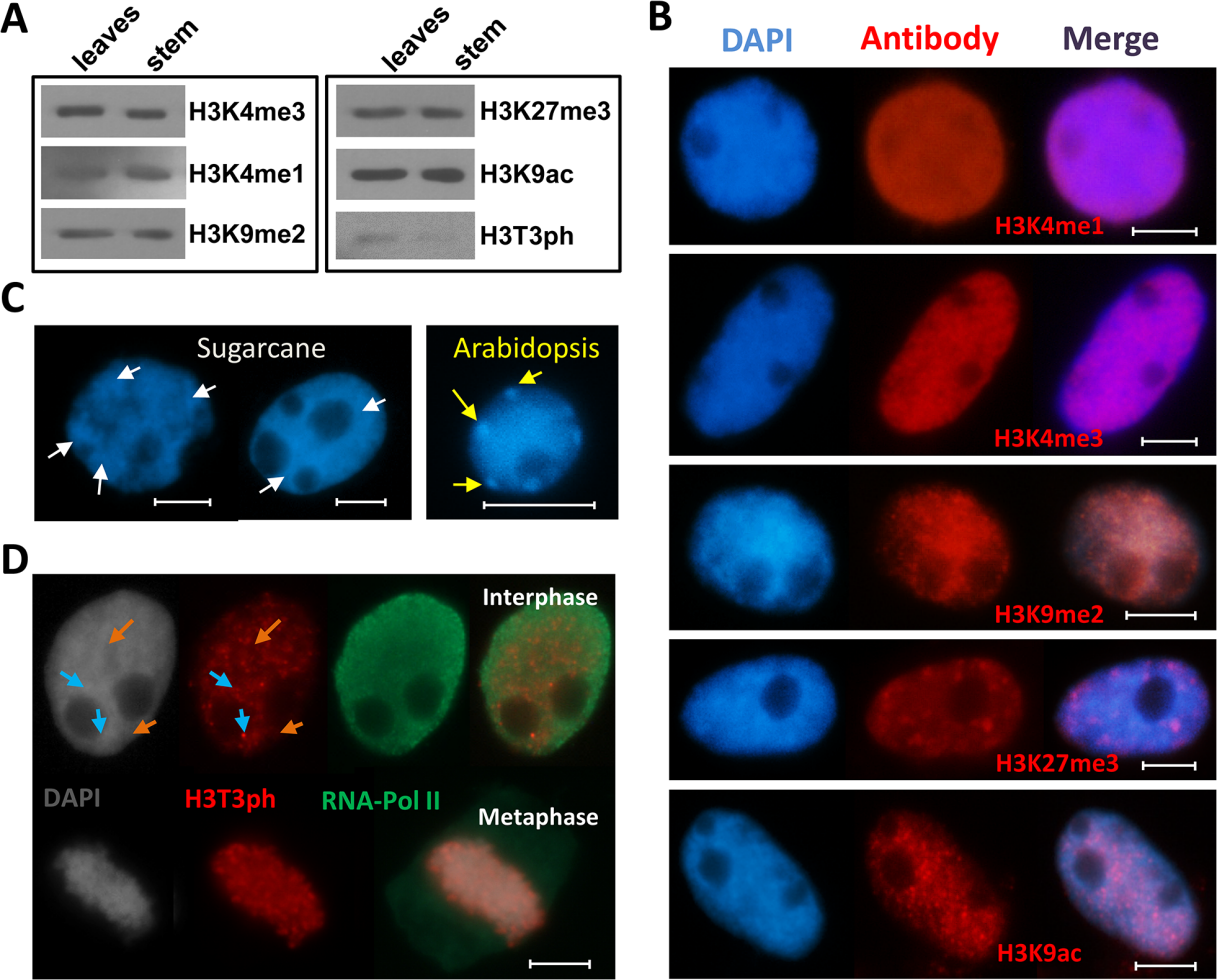
Distribution patterns of histone post-translational modifications in sugarcane. (A) Immunoblot analysis of global histone H3 modifications in sugarcane tissues. (B) Sub-nuclear localization of H3K4me1, H3K4me3, H3K9me2, H3K27me3 and H3K9ac. (C) Chromatin distribution of sugarcane and Arabidopsis; white arrows show DAPI densely stained regions in sugarcane, representing heterochromatic blocks. In Arabidopsis, the chromocenters are well defined regions of heterochromatin (yellow arrows). (D) H3T3ph (red signals) does not co-localize with actively transcribed regions rich in RNA Polymerase II (green signals). Instead, it appears to be associated with silent chromatin; DAPI densely stained regions (grey nucleus, blue arrows) coincide with H3T3ph brighter foci (red nucleus, blue arrows), whereas weaker/absent H3T3ph regions (red nucleus, orange arrows) coincide with the less condensed chromatin poorly stained with DAPI (grey nucleus, orange arrows). Bars = 5 μm.

To investigate the conservation of nuclear distribution of histone modifications between sugarcane and other plant species, we applied immunostaining using antibodies against H3K4me1, H3K4me3, H3K9me2, H3K27me3, H3K9ac and H3T3ph in nuclei isolated from root cells of sugarcane. Prior to immunostaining, we confirmed the presence of these modifications and the specificity of the antibodies on purified histones from sugarcane leaves and stems. Immunoblot analysis indicates that H3K4me1, H3K4me3, H3K9me2, H3K27me3 and H3K9ac, modifications previously identified by nanoLC-MS/MS, are indeed present in sugarcane histones from leaves and stems. Although we could not confidently detect histone phosphorylation in our analysis we decided to use a site specific antibody to analyze this modification. In plants, phosphorylation at serine residues is associated with the pericentromeric region, while threonine phosphorylation has been linked to chromosome condensation. Because these roles are conserved among all the plants studied so far, we decided to use an antibody against H3T3ph to investigate its localization in sugarcane nuclei (Fig 10).

The distribution of most methylation marks in histone residues of sugarcane are in general conserved with other organisms. All methylation states of H3K4, a universal mark for open chromatin, are confined to the euchromatin of sugarcane nuclei (Fig 10B). In contrast, H3K9me2, a mark for constitutive heterochromatin, is dispersed throughout the nuclei (Fig 10B). In Arabidopsis, highly condensed chromatin is easily distinguished in interphase nuclei as DAPI-positive domains, the chromocenters [43]. In sugarcane, DAPI densely stained domains are more widely spread (Fig 10C). H3K9me2 co-localizes with these regions, but it seems to be also present in the euchromatic regions (Fig 10B).

H3K27me3 is associated with euchromatin in Arabidopsis and barley. In *Vicia faba*, H3K27me3 signals were found associated with heterochromatin. In sugarcane, signals are distributed over the nucleus with some brighter foci. These foci do not fully co-localize with DAPI-positive regions indicating that in sugarcane this modification is linked to euchromatin. Further experiments will be necessary to find out whether H3K27me3 can also associate with heterochromatin.

We next tested the localization of H3K9ac, a mark linked to accessible chromatin. Consistent with that, H3K9ac signals are dispersed over the nuclei, with some stronger signals that do not co-localize with the heterochromatin densely stained by DAPI (Fig 10B).

In contrast to other plant species, in which H3T3ph is not detectable during interphase (reviewed in [24]), H3T3ph is localized to distinct domains within the interphase nucleus of sugarcane (Fig 10D). H3T3ph distribution includes several small foci throughout the nucleoplasm, some of these foci co-localizing with regions more densely stained with DAPI. To find out whether these foci co-localize with transcriptionally active regions, we applied double immunostaining using antibodies against RNA Polymerase II and H3T3ph (Fig 10D). Areas strongly stained with H3T3ph are mostly confined to areas where RNA polymerase II fluorescence is weaker or absent, indicating that during interphase H3T3ph is indeed a modification associated with non-transcribed regions. Strong H3T3ph signals were found associated with metaphase chromosomes, further suggesting that also in sugarcane there is an association of H3T3ph with chromosome condensation during cell division (Fig 10D).

## Discussion

### Histone H3 and H4 genes in sugarcane

Blast searches in the sugarcane EST collection allowed us to identify several homologs of the histones H3 and H4. In Arabidopsis, histone H3 proteins fall into two categories; the canonical histone H3.1, and the histone H3 variants including the replication-independent or replacement histone variant H3.3 and CENH3 [44]. The canonical H3.1 differs from the H3.3 variant only in four amino acids, A31T, F41Y, S87H and A90L whereas CENH3 is a more specialized and divergent variant [6, 17, 44]. Based on amino acid sequence, sugarcane histone H3 proteins were classified into canonical histone H3 (Ss_H3.1) and two variants; the replacement histone Ss_H3.3 and two isoforms of CENH3 (Ss_CENH3a and b). For histone H4, the proteins encoded were identical to the canonical H4 with the exception of a variant with a single amino acid change. Ss_H3.1, Ss_H3.3 and Ss_H4.1 are identical to their orthologs in other plant species including Arabidopsis, rice and maize. This is not surprising considering that histone H3 and H4 are among the most slowly evolving eukaryotic proteins. However, as observed in Arabidopsis and sugarcane, variants with specialized functions are common in H3 while histone H4 remains almost invariable. This contrasting functional specialization of the H3-H4 pair is common throughout eukaryote evolution [40].

In metazoans, histones are present in multigene families usually organized in clusters. In plants, histone genes are also present in multiple copies, but these are dispersed throughout the genome [45]. There are 15 histone H3 genes in the Arabidopsis genome, five H3.1, three H3.3, five H3.3 like, one CENH3 and one male gametic variant [44]. The Arabidopsis genome also encodes 8 histone H4 genes. Since our searched was limited to EST clusters representing transcripts it is difficult to determine if the different SAS identified comes from different loci or are allelic forms of the same gene. However, the ploidy level in sugarcane (which varies from 10–12x) and the high number of SAS per histone type (ranging from 17–20) reveals that there are at least two copies of each gene in Sugarcane. Although the number of histone genes in sugarcane could be significantly larger, the lack of genome sequence matching the SASs makes it difficult to assess the presence of introns and other genomic features that may help to differentiate between genes and alleles.

### Histone modifications in sugarcane

Histones are the main structural components of the chromatin. As such they are targets of many modifying enzymes that mediate processes involving changes in chromatin structure. Here, using a combination of mass spectrometry and modification-specific antibodies, we identified and analyzed PTMs in the histones H3 and H4 from sugarcane. Mass spectrometry allowed us to map different types of modifications to their respective residues and to determine their relative abundance whereas immunostaining with modification-specific antibodies allow us to visualize the sub-nuclear distributions of different modifications. To our knowledge, this is the first comprehensive catalog of histone modifications in H3 and H4 histones of any monocot species.

Histone modifications, namely acetylation, methylation and phosphorylation, in sugarcane H3 and H4 were found in many sites previously described in Arabidopsis and soybean. In addition, novel modifications and residues, not described before in plants, were identified particularly in the globular domain. The function of many modifications is evolutionary conserved in eukaryotes whereas others may be lineage specific. For instance, H3K4 methylation appears to be a universal mark for transcription in almost all eukaryotes studied including plants [46]. In contrast, H3K9me3 is a mark associated to heterochromatin in metazoans whereas in plants is H3K9me2 [46]. To obtain clues about conservation of PTMs in sugarcane we decided to study their sub-nuclear distribution using immunostaining. High conservation between sugarcane and other plant species was found in the distribution of most modifications studied (H3K4me1, H3K4me3, H3K9ac and H3K27me3). One exception was H3K9me2, however, this modification has been reported to display variation according to the genome size [14]. H3T3ph represents another exception, it was shown to be present in the interphase nuclei in sugarcane but was absent from this stage in Arabidopsis. This initial study indicates that the localization (and consequently the function) of the tested histone PTMs is mainly conserved in sugarcane. Nevertheless, to elucidate the function and organization of chromatin in sugarcane, further studies to map the chromatin and chromosome structure, possibly applying fluorescence *in situ* hybridization in combination with immunostaining, will be essential.

In general, we found that lysine acetylation is restricted to the N-terminal tails of histone H3 and H4 whereas lysine methylation, in addition to the tail, also occurred in the globular domain. The reason for the bias in the distribution of acetylation is currently unknown. Lysine acetylation may be present in the core domain but in levels below the detection limit, or may be regulated in a tissue specific fashion. Modifications of particular residues may be more frequent in the tails because they are more accessible than the more restricted globular regions that interact with the DNA. However, acetylation of residues corresponding to the core domain of histone H3 and H4 has been identified in other organisms indicating that lysine acetylation does occur in the globular domain [47]. In plants, two studies that aimed to identify histone modifications in Arabidopsis and soybean also failed to detect acetylated residues in the globular domain of histones H3 and H4 but not in the tail domain [18, 20]. In Arabidopsis, the presence of H3K56ac has been demonstrated using modification specific antibodies. Indeed, H3K56ac is a euchromatic histone modification associated with expressed genes and enriched at promoter regions [19, 48]. H3K56ac has also been associated with DNA replication [49]. Although the levels of H3K56ac are unknown, a significant amount of H3K56 is likely to be acetylated since ChIP on Chip analysis indicates H3K56ac covers approximately 34% of the genome [48]. Thus, it is possible that lysine residues in H3 and H4 globular domains, such as H3K56, are likely to be acetylated, but undetected with the methods used here.

During the nanoLC-MS/MS analysis of histone H3 and H4 from sugarcane, peptides containing O-acetylation in H3S10, H3T22, H3S28, H3Y41, H3Y54 and H4T30 were identified. Recently, O-acetylation of serine, threonine and tyrosine (S/T/Y) was described as a novel H3 modification present in several eukaryotes including yeast, Tetrahymena and metazoans [22]. Identification of S/T/Y O-acetylation in sugarcane extends the range of eukaryotic groups in which these modifications has been observed implicating that they are more evolutionary conserved than previously thought. This modification is usually found in very low abundance, ≤1%, in all the organisms studied [22]. Accordingly, H3S10ac and H3T22ac from sugarcane were also present at low relative abundance. The low levels of S/T/Y O-acetylation in sugarcane, and other organisms, indicates that these marks may be restricted to very specific genomic loci. Alternatively, like H3S10ac in humans, S/T/Y acetylation levels may be regulated in a cell type/cell cycle specific fashion or in response to certain stimuli. O-acetylation of H3S10 was proposed to function by blocking phosphorylation of the same residue thereby acting as an antagonist to H3S10ph [22]. Intriguingly, we found S/T/Y acetylation almost exclusively associate with lysine and arginine methylation. This observation suggests that O-acetylation of S/T/Y coexists with silencing marks such as methyl H3K9 and H3K27, but not with acetylated lysine, a mark usually associated to gene expression. The function of these modifications however still remains to be explored in plants and other organisms.

Comparison of the relative levels of some modifications between sugarcane and Arabidopsis reveals some similarities that suggest they may be functionally conserved. For instance, predominance of the acetylated over the methylated forms of H3K18 and H3K23 was observed in both Arabidopsis [17] and sugarcane. Also in both plants, methyl H3K9 and acetyl K14 are the major modifications found at residues 9–17, but only 2–3% of the peptides are doubly modified. The mutual exclusion of these modifications possibly occurs due to their antagonistic roles: H3K9me1/2 is associated with gene silencing at heterochromatic regions whereas H3K14ac is associated with gene expression at euchromatin. In Arabidopsis, HDA6 mediated deacetylation of H3K14, and other lysine residues, is necessary for the subsequent H3K9 methylation and for heterochromatic silencing [50, 51]. In a similar way, expression of ABA and salt-inducible genes leads to increased H3K9ac and H3K14ac and decreased H3K9me2 [52]. Exclusion of H3K9 methylation and H3K14ac from the same tail suggests that at least for these particular residues, the interplay between these modifications, and perhaps their functions, may be conserved in sugarcane.

The canonical histone H3.1 is associated to silent regions of the genome (i.e., regions containing methylation of H3K27, H3K9 and also DNA methylation). The replication independent H3.3 variant, on the other hand, is distributed along transcribed regions with a bias toward the 3’ end of genes [53, 54]. Consistent with its distribution, H3.3 has been shown to be enriched in modifications associated to gene activation. In Arabidopsis, H3.1 was found to contain higher levels of the silencing modification (H3K27 methylation) whereas H3.3 was enriched in K36 methylation, a mark associated with transcriptional activity [17]. In the same way, in soybean H3K36 methylation could be easily detected in histone H3.3, but not in H3.1 [20]. In sugarcane, we observed similar differences between these two modifications in Ss_H3.1 and Ss_H3.3. The levels of methyl H3K27, especially H3K27me3, were consistently higher in Ss_H3.1 than Ss_H3.3. In contrast, Ss_H3.1 showed low levels of H3K36me2/3 (3% in total) compared to the 13% found in Ss_H3.3. Surprisingly, we found high levels of H3K36me1 (37%) in Ss_H3.1 whereas this modification was not detected at all in Arabidopsis H3.1. In Arabidopsis and rice it has been shown that although H3K36 methylation is a euchromatic mark, only H3K36me2/3 are associated with transcriptional activity [55–58]. In addition, H3K36me1 and H3K36me2/3 appear to be catalyzed by different enzymes in Arabidopsis [55]. Thus, it is likely that H3K36me1 in Ss_H3.1 may index euchromatic regions not particularly associated to transcriptional activity. Overall, these observations indicate that, similarly to Arabidopsis, the sugarcane replacement histone Ss_H3.3 is enriched in H3K36me2/3, modifications associated with transcriptional activity.

For some modifications such as H3K9me2 and H3T3ph, clear differences from other species regarding nuclear distribution and/or relative amounts were observed. Considering that the genomes of Arabidopsis and sugarcane are very contrasting, these differences are likely the result of variations in genome organization and architecture. The interphase nuclei of Arabidopsis (2n = 10) display heterochromatic blocks, the chromocenters, that mark the position of each centromere and of the nucleolus-organizing regions of chromosomes 2 and 4 [43, 59]. Chromocenters contain most of the repetitive DNA sequences, constituting 15% of the Arabidopsis genome that cluster in the pericentromeric regions and the heterochromatic knobs. In contrast, the sugarcane genome is 10,000 Mbp, 50 times larger to that of Arabidopsis, is polyploid (10–12x) and aneuploid with between 100 to 130 chromosomes [60, 61]. The fraction, pattern and composition of heterochromatin in sugarcane are currently unknown. From preliminary BAC sequencing (covering 3.7% of the genome) it was calculated that at least 50% of the sequences are repetitive elements including transposable elements and satellite repeats [62]. These transposons are organized in complex arrays and insertions that are found flanking genes [62, 63]. Based on these observations, we expect a large fraction, at least ~50%, of the sugarcane genome to be heterochromatic. This fraction will be organized as small heterochromatic domains interspersed with euchromatin. The diffuse DAPI staining of chromatin in sugarcane interphase root nuclei supports this idea.

In several plant species, H3K9me1 and H3K9me2 are heterochromatic marks associated with silent repeats and transposons. In Arabidopsis, MS/MS analysis indicates that 10% of histone H3 is dimethylated at H3K9, likely a reflection of the amount of heterochromatin in the genome [17]. Interestingly, the significantly higher proportion of H3K9me2 (~40%) in sugarcane matches the proportion of repeats (~50%) which could be a close estimation of the heterochromatic fraction. H3K9me2 has been reported to display variation according to the genome size [14]. In species with a genome size smaller than 500mbp/1C, H3K9me2 signals were restricted to clustered heterochromatic regions while in species with larger genome size, all the nuclei were stained. Increase in genome size is usually the result of increased number of transposons, and consequently of heterochromatin, within intergenic regions [64]. Thus, the interspersed heterochromatin displaying diffuse nuclear distribution of H3K9me2, the mark associated to it, is an expected consequence of sugarcane genome size.

## Supporting Information

S1 Fig Amino acid sequence alignment of histone H3 from sugarcane and other plant species. Four amino acid changes between H3.1 and H3.3 from the different plant species are shown in blue and black (positions 31, 41, 87, 90). In the consensus line "*" indicates positions which have a fully conserved residue and ":" indicates 'strong' conserved groups (amino acids STA or FYW). Species are designated by a two-letter abbreviation preceding the name of each protein: At, *Arabidopsis thaliana*; Os, *Oryza sativa*; Ss, *Saccharum sp* var SP80-3280; Zm, *Zea mays*. Accession numbers of Ss_H3.1 and Ss_H3.2 are given in S1 Table. Accession numbers of proteins used in the analysis are as follows: At_HTR2, NP_563838; At_HTR4, NP_001078516; Os_HTR704, NP_001065791; Os_HTR711, NP_001050276; Zm_HTR102, XP_008659340.1; Zm_HTR105, XP_008659267.
(PDF)

S2 Fig Analysis of CENH3 proteins from sugarcane and other organisms. (Figure A) Multiple sequence alignment of sugarcane CENH3 isoforms and other plants. Amino acid differences between Ss_CENH3.a and b are shown with a blue shade. A C-terminal threonine (T) residue conserved in all CENH3 proteins and Ss_H3.3 is shown in red. The degree of conservation is distinguished at three levels (100, 80, and not conserved), where 100% has the darkest shade of the grey. (Figure B) Phylogenetic three showing the relationship between CENH3 from sugarcane and other organisms. Numbers on the nodes correspond to percentage bootstrap values based on 1000 pseudoreplicates. Only values higher than 60% are shown. Species are designated by a two-letter abbreviation preceding the name of each protein: At, *Arabidopsis thaliana*; Bd, *Brachypodium distachyon*; Hv, *Hordeum vulgare*; Ln, *Luzula nivea*; Os, *Oryza sativa*; Ss, *Saccharum sp* var SP80-3280; Sb, *Sorghum bicolor*; Zm, *Zea mays*. Accession numbers of Ss_H3.3, Ss_CENH3.a and b are given in S1 Table. Accession numbers of proteins used in the analysis are as follows: At_HTR12, NP_001030927; Bd_CENH3, XP_003566107; Hv_CENH3.a, AEK21392; Hv_CENH3.b, AEK21393; Ln_CENH3.a, BAE02657; Ln_CENH3.b, ADM18965; Os_CENH3, AAR85315; Sb_CENH3, XP_002441290; Zm_CENH3, NP_001105520.
(PDF)

S3 Fig Amino acid sequence alignment of histone H4 from sugarcane and other plant species. The Y72C change between Ss_H4.2 and the other H4 proteins is indicated in blue and black respectively. In the consensus line "*" indicates positions which have a fully conserved residue. Species are designated by a two-letter abbreviation preceding the name of each protein: At, *Arabidopsis thaliana*; Os, *Oryza sativa*; Ss, *Saccharum sp* var SP80-3280; Zm, *Zea mays*. Accession numbers of Ss_H4.1 and Ss_H4.2 are given in S2 Table. Accession numbers of proteins used in the analysis are as follows: At_HFO1, NP_850660; Os_HFO701, NP_001065179; Zm_HFO102, XP_008653962.
(PDF)

S4 Fig SDS-PAGE analysis of histone preparations from sugarcane. Histones were prepared from nuclei isolated from leaf roll tissues and further purified using the cation exchange resin Bio-Rex 70. Core histones were labeled according to size and by comparison with published histone preparations from cauliflower [31] and alfalfa [41].
(PDF)

S5 Fig MS/MS fragmentation pattern recorded on the [M+2H]^2+^ ion at *m*/*z* 556.3089 that matches the histone H3 peptide (residues 9–17) prKme1SacTGGKacAPR containing monomethyl K9, acetyl S10 and acetyl K14. Sequence of the peptide and the measured mass of the precursor ion are shown in the figure inset. N-terminal propionylation, product of the chemical derivatization, is indicated by pr.
(TIF)

S6 Fig MS/MS spectra of the doubly-charged precursor ion at *m*/*z* 598.8534 matching the H3 peptide prKacQLATacKme1AAR (residues 18–26) containing K18ac, T22ac and K23me1. Sequence of the modified peptide and the measured mass of the precursor ion are shown in the figure inset. N-terminal propionylation, product of the chemical derivatization, is indicated by pr.
(TIF)

S7 Fig Sequence coverage of sugarcane Ss_CENH3.a and Ss_CENH3.b by the peptides identified in the nanoLC-MS/MS analysis. The positions of the peptides identified are shown in blue bars below the protein sequence. Amino acids matching the peptide sequence are indicated in red. The coverage for Ss_CENH3.a and Ss_CENH3.b is 29.5% and 34.6% respectively.
(PDF)

S1 Table List of Sugarcane Assembled Sequences (SAS) encoding sugarcane histone H3. (PDF)

S2 Table List of Sugarcane Assembled Sequences (SAS) encoding sugarcane histone H4. (PDF)

S3 Table List of modified peptides corresponding to sugarcane histone H3 identified in the nanoLC-MS/MS analysis of bulk histones. (PDF)

S4 Table List of modified peptides corresponding to sugarcane histone H4 identified in the nanoLC-MS/MS analysis of bulk histones. (PDF)
