# Supplementary material for: Analysis of Histones H3 and H4 Reveals Novel and Conserved Post-Translational Modifications in Sugarcane
Source: PLoS One. 2015 Jul 30;10(7):e0134586. doi: 10.1371/journal.pone.0134586 (PMC4520453; doi:10.1371/journal.pone.0134586)
Supplement: S1 Fig — Four amino acid changes between H3.1 and H3.3 from the different plant species are shown in blue and black (positions 31, 41, 87, 90). In the consensus line "*" indicates positions which have a fully conserved residue and ":" indicates 'strong' conserved groups (amino acids STA or FYW). Species are designated by a two-letter abbreviation preceding the name of each protein: At, Arabidopsis thaliana; Os, Oryza sativa; Ss, Saccharum sp var SP80-3280; Zm, Zea mays. Accession numbers of Ss_H3.1 and Ss_H3.2 are given in S1 Table. Accession numbers of proteins used in the analysis are as follows: At_HTR2, NP_563838; At_HTR4, NP_001078516; Os_HTR704, NP_001065791; Os_HTR711, NP_001050276; Zm_HTR102, XP_008659340.1; Zm_HTR105, XP_008659267. (PDF) [file pone.0134586.s001.pdf]

```
Ss_H3.1    :ARTKQTARKSTGGKAPRKQLATKAARKSAPATGGVKKPHRFRPGTVALREIRKYQKSTELLIRKLPFQRL : 70
At_HTR2    :ARTKQTARKSTGGKAPRKQLATKAARKSAPATGGVKKPHRFRPGTVALREIRKYQKSTELLIRKLPFQRL : 70
Os_HTR704  :ARTKQTARKSTGGKAPRKQLATKAARKSAPATGGVKKPHRFRPGTVALREIRKYQKSTELLIRKLPFQRL : 70
Zm_HTR105  :ARTKQTARKSTGGKAPRKQLATKAARKSAPATGGVKKPHRFRPGTVALREIRKYQKSTELLIRKLPFQRL : 70
Ss_H3.3    :ARTKQTARKSTGGKAPRKQLATKAARKSAPTTGGVKKPHRYRPGTVALREIRKYQKSTELLIRKLPFQRL : 70
At_HTR4    :ARTKQTARKSTGGKAPRKQLATKAARKSAPTTGGVKKPHRYRPGTVALREIRKYQKSTELLIRKLPFQRL : 70
Os_HTR711  :ARTKQTARKSTGGKAPRKQLATKAARKSAPTTGGVKKPHRYRPGTVALREIRKYQKSTELLIRKLPFQRL : 70
Zm_HTR102  :ARTKQTARKSTGGKAPRKQLATKAARKSAPTTGGVKKPHRYRPGTVALREIRKYQKSTELLIRKLPFQRL : 70
Consensus   ******************************:*********:*****************************

Ss_H3.1    :VREIAQDFKTDLRFQSSAVAALQEAAEAYLVGLFEDTNLCAIHAKRVTIMPKDIQLARRIRGERA : 135
At_HTR2    :VREIAQDFKTDLRFQSSAVAALQEAAEAYLVGLFEDTNLCAIHAKRVTIMPKDIQLARRIRGERA : 135
Os_HTR704  :VREIAQDFKTDLRFQSSAVAALQEAAEAYLVGLFEDTNLCAIHAKRVTIMPKDIQLARRIRGERA : 135
Zm_HTR105  :VREIAQDFKTDLRFQSSAVAALQEAAEAYLVGLFEDTNLCAIHAKRVTIMPKDIQLARRIRGERA : 135
Ss_H3.3    :VREIAQDFKTDLRFQSHAVLALQEAAEAYLVGLFEDTNLCAIHAKRVTIMPKDIQLARRIRGERA : 135
At_HTR4    :VREIAQDFKTDLRFQSHAVLALQEAAEAYLVGLFEDTNLCAIHAKRVTIMPKDIQLARRIRGERA : 135
Os_HTR711  :VREIAQDFKTDLRFQSHAVLALQEAAEAYLVGLFEDTNLCAIHAKRVTIMPKDIQLARRIRGERA : 135
Zm_HTR102  :VREIAQDFKTDLRFQSHAVLALQEAAEAYLVGLFEDTNLCAIHAKRVTIMPKDIQLARRIRGERA : 135
Consensus   **************** ** *********************************************
```
