# Supplementary material for: Analysis of Histones H3 and H4 Reveals Novel and Conserved Post-Translational Modifications in Sugarcane
Source: PLoS One. 2015 Jul 30;10(7):e0134586. doi: 10.1371/journal.pone.0134586 (PMC4520453; doi:10.1371/journal.pone.0134586)
Supplement: S2 Fig — (Figure A) Multiple sequence alignment of sugarcane CENH3 isoforms and other plants. Amino acid differences between Ss_CENH3.a and b are shown with a blue shade. A C-terminal threonine (T) residue conserved in all CENH3 proteins and Ss_H3.3 is shown in red. The degree of conservation is distinguished at three levels (100, 80, and not conserved), where 100% has the darkest shade of the grey. (Figure B) Phylogenetic three showing the relationship between CENH3 from sugarcane and other organisms. Numbers on the nodes correspond to percentage bootstrap values based on 1000 pseudoreplicates. Only values higher than 60% are shown. Species are designated by a two-letter abbreviation preceding the name of each protein: At, Arabidopsis thaliana; Bd, Brachypodium distachyon; Hv, Hordeum vulgare; Ln, Luzula nivea; Os, Oryza sativa; Ss, Saccharum sp var SP80-3280; Sb, Sorghum bicolor; Zm, Zea mays. Accession numbers of Ss_H3.3, Ss_CENH3.a and b are given in S1 Table. Accession numbers of proteins used in the analysis are as follows: At_HTR12, NP_001030927; Bd_CENH3, XP_003566107; Hv_CENH3.a, AEK21392; Hv_CENH3.b, AEK21393; Ln_CENH3.a, BAE02657; Ln_CENH3.b, ADM18965; Os_CENH3, AAR85315; Sb_CENH3, XP_002441290; Zm_CENH3, NP_001105520. (PDF) [file pone.0134586.s002.pdf]

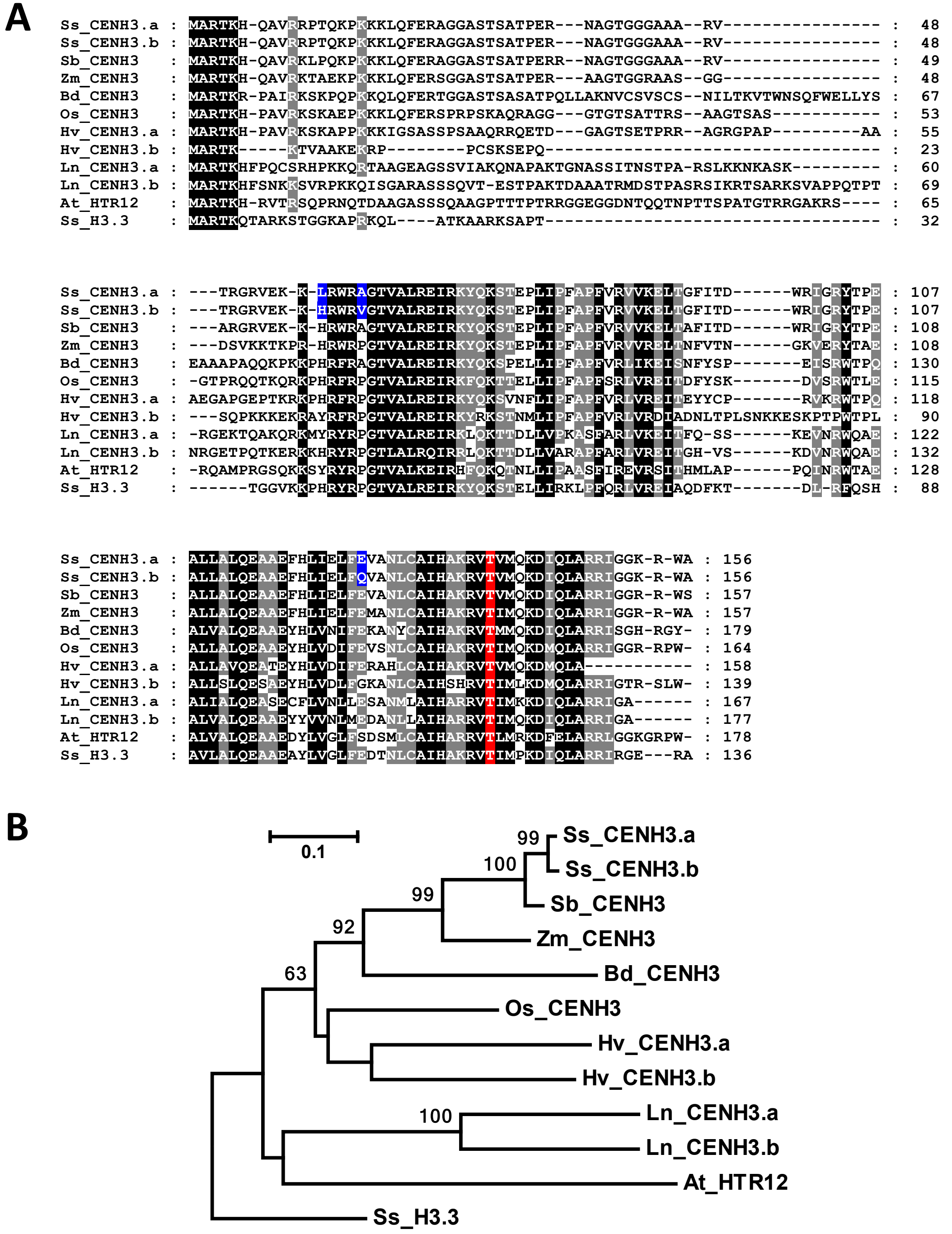
A
Ss_CENH3.a : MARTKH-QAVRRPTQKPKKKLQFERAGGASTSATPER---NAGTGGGAAA--RV--------------- : 48
Ss_CENH3.b : MARTKH-QAVRRPTQKPKKKLQFERAGGASTSATPER---NAGTGGGAAA--RV--------------- : 48
Sb_CENH3 : MARTKH-QAVRKLPQKPKKKLQFERAGGASTSATPERR--NAGTGGGAAA--RV--------------- : 49
Zm_CENH3 : MARTKH-QAVRKTAEKPKKKLQFERSGGASTSATPER---AAGTGGRAAS--GG--------------- : 48
Bd_CENH3 : MARTKR-PAIRKSKPQPKKQLQFERTGGASTSASATPQLLAKNVCSVSCS--NILTKVTWNSQFWELLYS : 67
Os_CENH3 : MARTKH-PAVRKSKAEPKKKLQFERSPRPSKAQRAGG---GTGTSATTRS--AAGTSAS---------- : 53
Hv_CENH3.a : MARTKH-PAVRKSKAPPKKKIGSASSPSAAQRRQETD---GAGTSETPRR--AGRGPAP---------AA : 55
Hv_CENH3.b : MARTK-----KTVAAKEKRP--------PCSKSEPQ--------------------------------- : 23
Ln_CENH3.a : MARTKHFPQCSRHPKKQRTAAGEAGSSVIAKQNAPAKTGNASSITNSTPA-RSLKKNKASK--------- : 60
Ln_CENH3.b : MARTKHFSNKKSVRPKKQISGARASSSQVT-ESTPAKTDAAATRMDSTPASRSIKRTSARKSVAPPQTPT : 69
At_HTR12 : MARTKH-RVTRSQPRNQTDAAGASSSQAAGPTTTPTRRGGEGGDNTQQTNPTTSPATGTRRGAKRS---- : 65
Ss_H3.3 : MARTKQTARKSTGGKAPRKQL----ATKAARKSAPT--------------------------------- : 32
Ss_CENH3.a : ---TRGRVEK-K-LRWRAGTVALREIRKYQKSTEPLIPFAPFVRVVKELTGFITD------WRIGRYTPE : 107
Ss_CENH3.b : ---TRGRVEK-K-HRWRVGTVALREIRKYQKSTEPLIPFAPFVRVVKELTGFITD------WRIGRYTPE : 107
Sb_CENH3 : ---ARGRVEK-K-HRWRAGTVALREIRKYQKSTEPLIPFAPFVRVVKELTAFITD------WRIGRYTPE : 108
Zm_CENH3 : ---DSVKKTKPR-HRWRPGTVALREIRKYQKSTEPLIPFAPFVRVVRELTNFVTN------GKVERYTAE : 108
Bd_CENH3 : EAAAPAQQKPKKPHRFRAGTVALREIRKYQKSPELLIPFAPFVRLIKEISNFYSP-------EISRWTPQ : 130
Os_CENH3 : -GTPRQQTKQRKPHRFRPGTVALREIRKFQKTTELLIPFAPFSRLVREITDFYSK-------DVSRWTLE : 115
Hv_CENH3.a : AEGAPGEPTKRKPHRFRPGTVALREIRKYQKSVNFLIPFAPFVRLVREITEYYCP-------RVKRWTPQ : 118
Hv_CENH3.b : ---SQPKKKEKRAYRFRPGTVALREIRKYRKSTNMLIPFAPFVRLVRDIADNLTPLSNKKESKPTPWTPL : 90
Ln_CENH3.a : -RGEKTQAKQRKMYRYRPGTVALREIRKLQKTTDLLVPKASFARLVKEITFQ-SS------KEVNRWQAE : 122
Ln_CENH3.b : NRGETPQTKERKKHRYRPGTLALRQIRRLQKTTDLLVARAPFARLVREITGH-VS------KDVNRWQAE : 132
At_HTR12 : -RQAMPRGSQKKSYRYRPGTVALKEIRHFQKQTNLLIPAASFIREVRSITHMLAP------PQINRWTAE : 128
Ss_H3.3 : ------TGGVKKPHRYRPGTVALREIRKYQKSTELLIRKLPFQRLVREIAQDFKT-------DL-RFQSH : 88
Ss_CENH3.a : ALLALQEAAEFHLIELFEVANLCAIHAKRVTVMQKDIQLARRIGGK-R-WA : 156
Ss_CENH3.b : ALLALQEAAEFHLIELFQVANLCAIHAKRVTVMQKDIQLARRIGGK-R-WA : 156
Sb_CENH3 : ALLALQEAAEFHLIELFEVANLCAIHAKRVTVMQKDIQLARRIGGR-R-WS : 157
Zm_CENH3 : ALLALQEAAEFHLIELFEMANLCAIHAKRVTIMQKDIQLARRIGGR-R-WA : 157
Bd_CENH3 : ALVALQEAAEYHLVNIFEKANYCAIHAKRVTMMQKDIQLARRISGH-RGY- : 179
Os_CENH3 : ALLALQEAAEYHLVDIFEVSNLCAIHAKRVTIMQKDMQLARRIGGR-RPW- : 164
Hv_CENH3.a : ALLAVQEATEYHLVDIFERAHLCAIHAKRVTVMQKDMQLA----------- : 158
Hv_CENH3.b : ALLSLQESAEYHLVDLFGKANLCAIHSHRVTIMLKDMQLARRIGTR-SLW- : 139
Ln_CENH3.a : ALIALQEASECFLVNLLESANMLAIHARRVTIMKKDIQLARRIGA------ : 167
Ln_CENH3.b : ALVALQEAAEYYVVNLMEDANLLAIHARRVTIMQKDIQLARRIGA------ : 177
At_HTR12 : ALVALQEAAEDYLVGLFSDSMLCAIHARRVTLMRKDFELARRLGGKGRPW- : 178
Ss_H3.3 : AVLALQEAAEAYLVGLFEDTNLCAIHAKRVTIMPKDIQLARRIRGE---RA : 136
B
0.1
99
100
99
92
63
100
Ss_CENH3.a
Ss_CENH3.b
Sb_CENH3
Zm_CENH3
Bd_CENH3
Os_CENH3
Hv_CENH3.a
Hv_CENH3.b
Ln_CENH3.a
Ln_CENH3.b
At_HTR12
Ss_H3.3
