# Supplementary material for: Analysis of Histones H3 and H4 Reveals Novel and Conserved Post-Translational Modifications in Sugarcane
Source: PLoS One. 2015 Jul 30;10(7):e0134586. doi: 10.1371/journal.pone.0134586 (PMC4520453; doi:10.1371/journal.pone.0134586)
Supplement: S3 Fig — The Y72C change between Ss_H4.2 and the other H4 proteins is indicated in blue and black respectively. In the consensus line "*" indicates positions which have a fully conserved residue. Species are designated by a two-letter abbreviation preceding the name of each protein: At, Arabidopsis thaliana; Os, Oryza sativa; Ss, Saccharum sp var SP80-3280; Zm, Zea mays. Accession numbers of Ss_H4.1 and Ss_H4.2 are given in S2 Table. Accession numbers of proteins used in the analysis are as follows: At_HFO1, NP_850660; Os_HFO701, NP_001065179; Zm_HFO102, XP_008653962. (PDF) [file pone.0134586.s003.pdf]

```
Ss_H4.1     :SGRGKGGKGLGKGGAKRHRKVLRDNIQGITKPAIRRLARRGGVKRISGLIYEETRGVLKIFLENVIRDAV: 70
Ss_H4.2     :SGRGKGGKGLGKGGAKRHRKVLRDNIQGITKPAIRRLARRGGVKRISGLIYEETRGVLKIFLENVIRDAV: 70
At_HFO3     :SGRGKGGKGLGKGGAKRHRKVLRDNIQGITKPAIRRLARRGGVKRISGLIYEETRGVLKIFLENVIRDAV: 70
Os_HFO711   :SGRGKGGKGLGKGGAKRHRKVLRDNIQGITKPAIRRLARRGGVKRISGLIYEETRGVLKIFLENVIRDAV: 70
Zm_HFO102   :SGRGKGGKGLGKGGAKRHRKVLRDNIQGITKPAIRRLARRGGVKRISGLIYEETRGVLKIFLENVIRDAV: 70
Consensus    **********************************************************************

Ss_H4.1     :TYTEHARRKTVTAMDVVYALKRQGRTLYGFGG : 102
Ss_H4.2     :TCTEHARRKTVTAMDVVYALKRQGRTLYGFGG : 102
At_HFO3     :TYTEHARRKTVTAMDVVYALKRQGRTLYGFGG : 102
Os_HFO711   :TYTEHARRKTVTAMDVVYALKRQGRTLYGFGG : 102
Zm_HFO102   :TYTEHARRKTVTAMDVVYALKRQGRTLYGFGG : 102
Consensus    * ******************************
```
