# Supplementary figures and images for: Analysis of Histones H3 and H4 Reveals Novel and Conserved Post-Translational Modifications in Sugarcane
Source: PLoS One. 2015 Jul 30;10(7):e0134586. doi: 10.1371/journal.pone.0134586 (PMC4520453; doi:10.1371/journal.pone.0134586)

Marker

Total histones

170 kDa

70 kDa

55 kDa

40 kDa

35 kDa

25 kDa

15 kDa

10 kDa

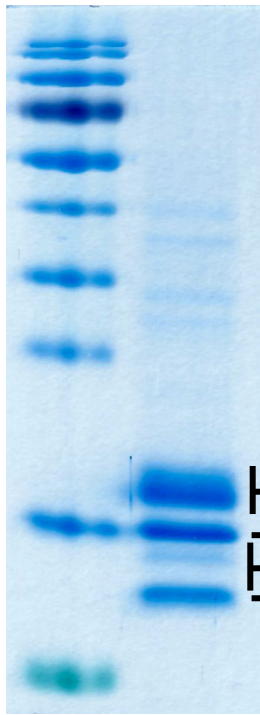

H2A/H2B4

H3

H2A/H2B4

H4

Supplement: S4 Fig — Histones were prepared from nuclei isolated from leaf roll tissues and further purified using the cation exchange resin Bio-Rex 70. Core histones were labeled according to size and by comparison with published histone preparations from cauliflower [31] and alfalfa [41]. (PDF) [file pone.0134586.s004.pdf]

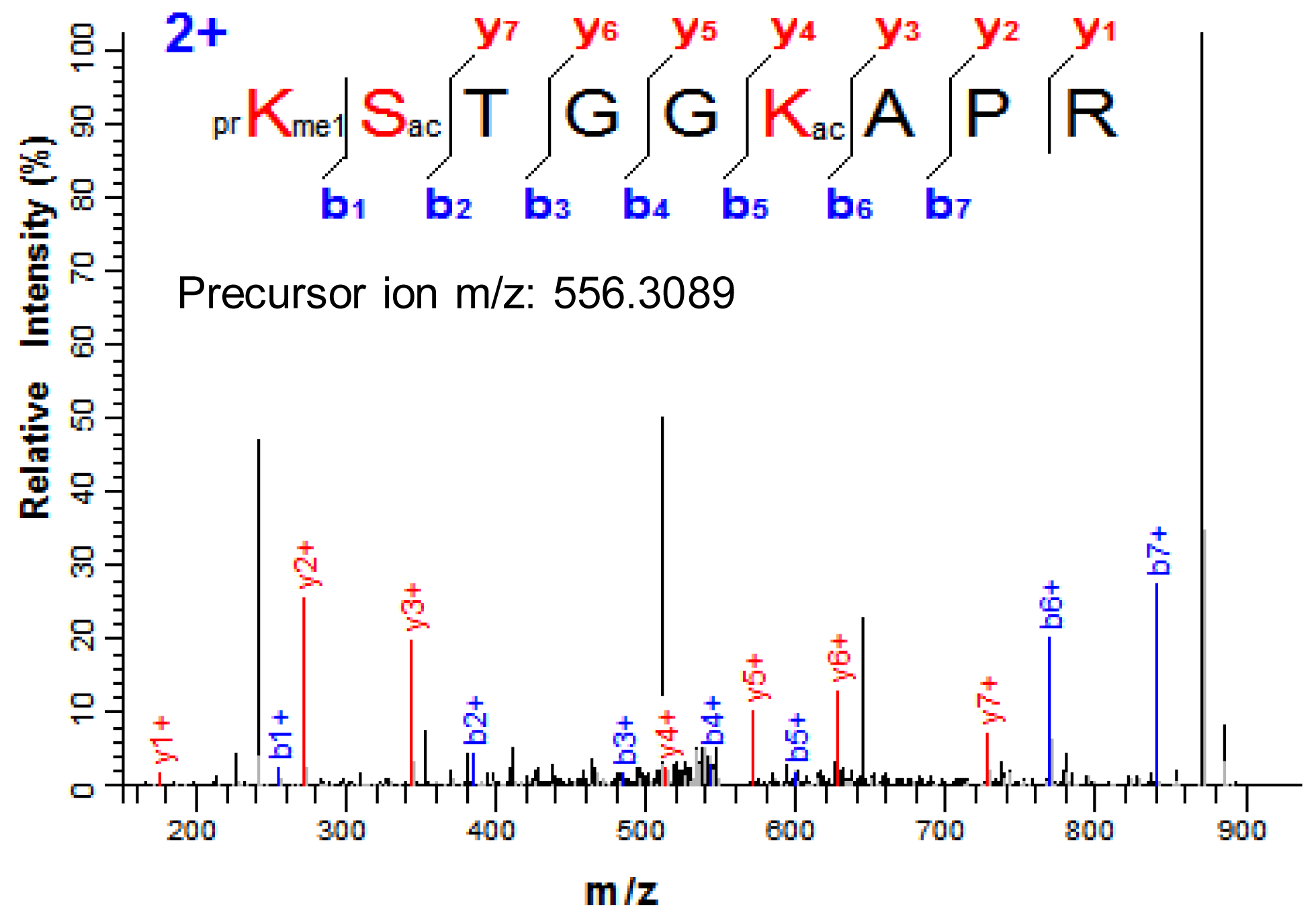

Supplement: S5 Fig — Sequence of the peptide and the measured mass of the precursor ion are shown in the figure inset. N-terminal propionylation, product of the chemical derivatization, is indicated by pr. (TIF) [file pone.0134586.s005.tif]

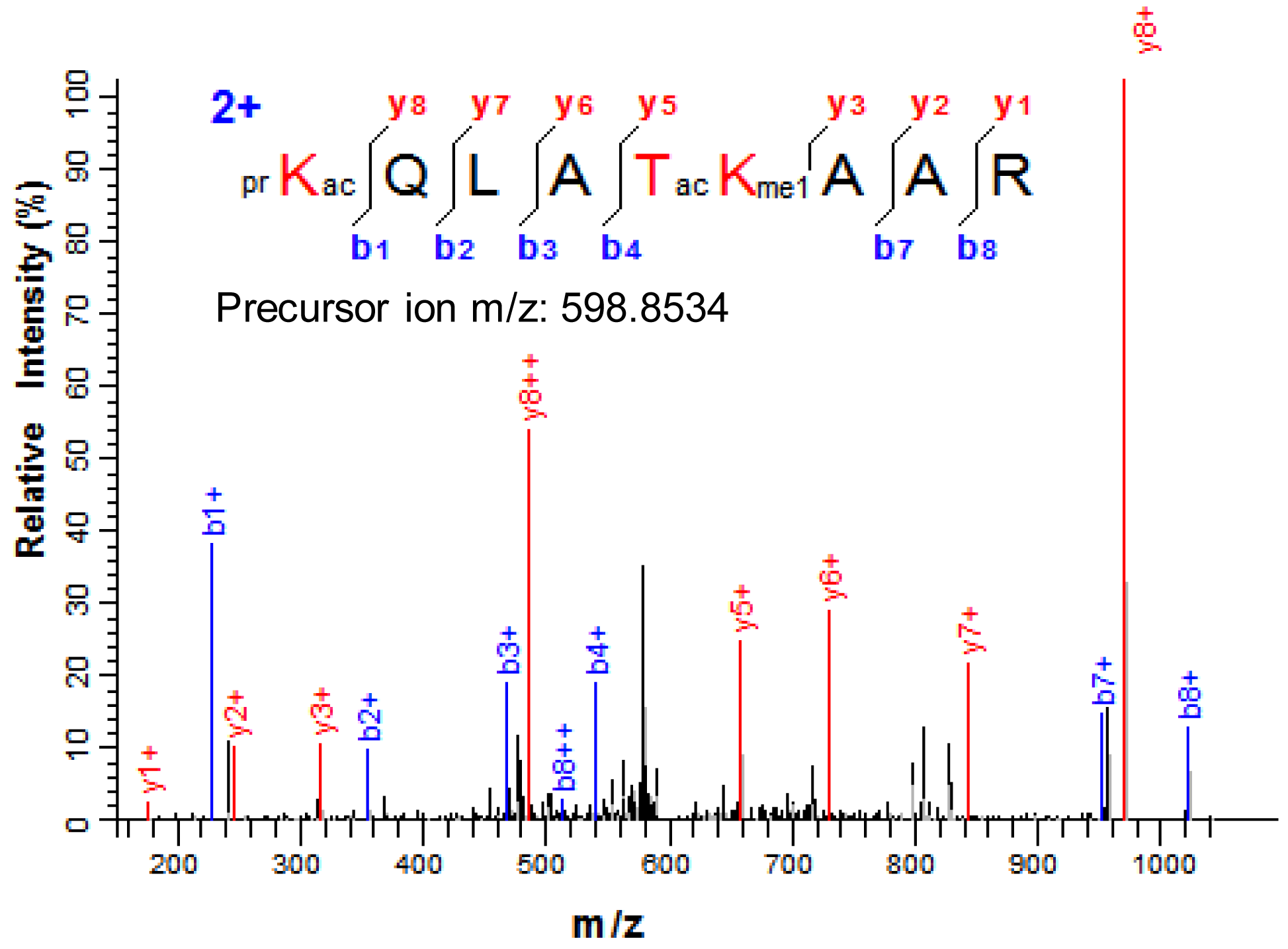

Supplement: S6 Fig — Sequence of the modified peptide and the measured mass of the precursor ion are shown in the figure inset. N-terminal propionylation, product of the chemical derivatization, is indicated by pr. (TIF) [file pone.0134586.s006.tif]
