# Supplementary material for: Analysis of Histones H3 and H4 Reveals Novel and Conserved Post-Translational Modifications in Sugarcane
Source: PLoS One. 2015 Jul 30;10(7):e0134586. doi: 10.1371/journal.pone.0134586 (PMC4520453; doi:10.1371/journal.pone.0134586)
Supplement: S7 Fig — The positions of the peptides identified are shown in blue bars below the protein sequence. Amino acids matching the peptide sequence are indicated in red. The coverage for Ss_CENH3.a and Ss_CENH3.b is 29.5% and 34.6% respectively. (PDF) [file pone.0134586.s007.pdf]

```
Ss_CenH3.a: ARTKHQAVRRPTQKPKKKLQFERAGGASTSATPERNAGTGGGAAARVTRGRVEKKLR : 57
Ss_CenH3.a: WRAGTVALREIRKYQKSTEPLIPFAPFVRVVKELTGFITDWRIGRYTPEALLALQEA :114
Ss_CenH3.a: AEFHLIELFEVANLCAIHAKRVTVMQKDIQLARRIGGKRWA : 155

Ss_CenH3.b: ARTKHQAVRRPTQKPKKKLQFERAGGASTSATPERNAGTGGGAAARVTRGRVEKKHR : 57
Ss_CenH3.b: WRVGTVALREIRKYQKSTEPLIPFAPFVRVVKELTGFITDWRIGRYTPEALLALQEA :114
Ss_CenH3.b: AEFHLIELFQVANLCAIHAKRVTVMQKDIQLARRIGGKRWA : 155
```
