# Supplementary material for: Analysis of Histones H3 and H4 Reveals Novel and Conserved Post-Translational Modifications in Sugarcane
Source: PLoS One. 2015 Jul 30;10(7):e0134586. doi: 10.1371/journal.pone.0134586 (PMC4520453; doi:10.1371/journal.pone.0134586)
Supplement: S1 Table — (PDF) [file pone.0134586.s008.pdf]

S1 Table. List of Sugarcane Assembled Sequences (SAS) encoding sugarcane histone H3.

Histone	SAS(a)	Accession number(b)	Protein	ORF
**H3.1**	SCEQLB1066C12.g	CA112618.1	Ss_H3.1	full
	SCCCLR1001F12.g	CA116174.1	Ss_H3.1	full
	SCAGLR2026E02.g	CA128090.1	Ss_H3.1	full
	SCAGFL8013E01.g	CA290173.1	Ss_H3.1	full
	SCCCLR1C04B07.g	CA189733.1	Ss_H3.1	full
	SCCCRZ2001G08.g	CA149659.1	Ss_H3.1	full
	SCVPLB1017H05.g	CA115839.1	Ss_H3.1	full
	SCJFRZ2009C12.g	CA151361.1	Ss_H3.1	full
	SCCCLR1068F12.g	CA120027.1	Ss_H3.1	full
	SCBFLR1026A06.g	CA117180.1	Ss_H3.1	full
	SCQGLR1062C03.g	CA124147.1	Ss_H3.1	full
	SCMCLR1032D12.g	CA123555.1	Ss_H3.1	full
	SCBGLR1002H11.g	CA117691.1	Ss_H3.1	full
	SCCCLR2001D10.g	CA127004.1	Ss_H3.1	full
	SCEQLB1063F02.g	CA112396.1	Ss_H3.1	full
	SCVPLB1016B08.g	CA115765.1	Ss_H3.1	full
	SCQGLR1019F06.g	CA124057.1	Ss_H3.1	full
**H3.3 variant**	SCBGLR1023F03.g	CA117709.1	Ss_H3.3	full
	SCEQLR1094C02.g	CA121383.1	Ss_H3.3	full
	SCUTLR2008H04.g	CA129793.1	Ss_H3.3	full
	SCCCRZ2002A05.g	CA149679.1	Ss_H3.3	full
	SCBGLR1002A05.g	CA117638.1	Ss_H3.3	full
	SCJFRZ2006G04.g	CA151144.1	Ss_H3.3	full
	SCJFLR1035H03.g	CA121848.1	Ss_H3.3	full
	SCCCCL3120E10.g	CA093669.1	Ss_H3.3	full
	SCCCFL5003D06.g	CA216595.1	Ss_H3.3	full
	SCBGFL3095F09.g	CA243335.1	Ss_H3.3	full
	SCJFLR1013C11.g	CA121755.1	Ss_H3.3	full
	SCRFLR1055C06.g	CA125316.1	Ss_H3.3	full
	SCCCLR2001F08.g	CA127024.1	Ss_H3.3	full
	SCVPRZ2039D03.g	CA154067.1	Ss_H3.3	full
	SCJFRT1060F02.g	CA134320.1	Ss_H3.3	full
	SCSGFL4037A02.g	CA222323.1	Ss_H3.3	truncated
	SCVPLR2027D12.g	CA130347.1	Ss_H3.3	truncated
	SCCCCL7C02A12.g	CA193522.1	Ss_H3.3	truncated
**CENH3**	SCCCLR2004A05.g	CA127217.1	Ss_CENH3.a	full
	SCCCLR1066H07.g	CA119873.1	Ss_CENH3.b	full
**H3 like**	SCQGFL1095C08.g	CA214738.1	H3.1_like	truncated
	SCQSRT2035A08.g	CA143377.1	H3.3_like	truncated
	SCRFLR1055A01.g	CA125283.1	H3.3_like	truncated

(a) Sugarcane Assembled Sequences correspond to unique transcripts assembled from a collection of ESTs developed by the SUCEST project (Vettore et al 2003) available at the sugarcane genome database (http://sucest-fun.org)

(b) GeneBank accession number corresponding to the longest EST after which individual SAS is named.
