# Supplementary material for: Analysis of Histones H3 and H4 Reveals Novel and Conserved Post-Translational Modifications in Sugarcane
Source: PLoS One. 2015 Jul 30;10(7):e0134586. doi: 10.1371/journal.pone.0134586 (PMC4520453; doi:10.1371/journal.pone.0134586)
Supplement: S2 Table — (PDF) [file pone.0134586.s009.pdf]

S2 Table. List of Sugarcane Assembled Sequences (SAS) encoding sugarcane histone H4.

Histone	SAS(a)	Accession number(b)	Protein	ORF
**H4 core**	SCCCRZ2001G01.g	CA149652.1	Ss_H4.1	full
	SCAGLB1070C07.g	CA111434.1	Ss_H4.1	full
	SCJLLR1011E11.g	CA122309.1	Ss_H4.1	full
	SCCCLR1078H02.g	CA120539.1	Ss_H4.1	full
	SCEPLR1051H12.g	CA120939.1	Ss_H4.1	full
	SCAGLB1069E02.g	CA111378.1	Ss_H4.1	full
	SCCCLR2002D04.g	CA127099.1	Ss_H4.1	full
	SCJFLR1013A12.g	CA121749.1	Ss_H4.1	full
	SCCCRZ1004C05.g	CA147082.1	Ss_H4.1	full
	SCSBFL1043F08.g	CA200936.1	Ss_H4.1	full
	SCSBLB1033H09.g	CA115422.1	Ss_H4.1	full
	SCCCLR2002G09.g	CA127138.1	Ss_H4.1	full
	SCCCLR2003E01.g	CA127174.1	Ss_H4.1	full
	SCJFRZ2009F02.g	CA151387.1	Ss_H4.1	full
	SCVPRZ2035C06.g	CA153713.1	Ss_H4.1	full
	SCJLLR2020B06.g	CA128654.1	Ss_H4.1	full
	SCCCLR2001D12.g	CA127006.1	Ss_H4.1	full
	SCRFLR2038C05.g	CA129343.1	Ss_H4.1	full
	SCCCLR2001D01.g	CA126997.1	Ss_H4.1	full
	SCSGRT2062A10.g	CA145162.1	Ss_H4.2	full
**H4 like**	SCUTLR2015B06.g	CA129817.1	H4_like	insertion/deletion
	SCJLHR1028C12.g	CA106117.1	H4_like	deletion
	SCSGHR1071E06.g	CA109488.1	H4_like	deletion
	SCAGLR2026F05.g	CA128039.1	H4_like	truncated

(a) Sugarcane Assembled Sequences correspond to unique transcripts assembled from a collection of ESTs developed by the SUCEST project (Vettore et al 2003) available at the sugarcane genome database (http://sucest-fun.org)

(b) GeneBank accession numbers corresponding to the longest EST after which individual SAS is named.
