# Supplementary material for: Analysis of Histones H3 and H4 Reveals Novel and Conserved Post-Translational Modifications in Sugarcane
Source: PLoS One. 2015 Jul 30;10(7):e0134586. doi: 10.1371/journal.pone.0134586 (PMC4520453; doi:10.1371/journal.pone.0134586)
Supplement: S3 Table — (PDF) [file pone.0134586.s010.pdf]

S3 Table. List of modified peptides corresponding to sugarcane histone H3 identified in the nanoLC-MS/MS analysis of bulk histones.

Sequence	Measured *m/z*	Calculated *m/z*	Charge	ppm	Modification	Histone type	Score	Retention time (min)
3-TKQTAR-8	401.7245	401.7245	2	-0.06	K4(ac)	Ss_H3.1, Ss_H3.3	1.82E-01	23.03
3-TKQTAR-8	415.7401	415.7401	2	-0.19	K4(me1)	Ss_H3.1, Ss_H3.3	1.74E-02	29.01
3-TKQTAR-8	394.7348	394.7349	2	-0.14	K4(me2)	Ss_H3.1, Ss_H3.3	9.84E-02	17.16
3-TKQTAR-8	401.7426	401.7427	2	-0.13	K4(me3)	Ss_H3.1, Ss_H3.3	1.15E-01	17.16
9-KSTGGKAPR-17	528.2960	528.2958	2	0.43	K9(ac)	Ss_H3.1, Ss_H3.3	3.00E-03	29.10
9-KSTGGKAPR-17	542.3115	542.3115	2	0.05	K9(me1)	Ss_H3.1, Ss_H3.3	1.20E-03	33.76
9-KSTGGKAPR-17	521.3059	521.3062	2	-0.48	K9(me2)	Ss_H3.1, Ss_H3.3	1.84E-02	23.87
9-KSTGGKAPR-17	528.3133	528.3140	2	-1.25	K9(me3)	Ss_H3.1, Ss_H3.3	2.83E-01	23.64
9-KSTGGKAPR-17	556.3089	556.3089	2	-0.02	S10(ac)	Ss_H3.1, Ss_H3.3	5.12E-02	34.32
9-KSTGGKAPR-17	528.2960	528.2958	2	0.43	K14(ac)	Ss_H3.1, Ss_H3.3	3.00E-03	29.46
9-KSTGGKAPR-17	521.2877	521.2880	2	-0.60	K9(ac), K14(ac)	Ss_H3.1, Ss_H3.3	6.92E-02	27.70
9-KSTGGKAPR-17	535.3035	535.3036	2	-0.32	K9(me1), K14(ac)	Ss_H3.1, Ss_H3.3	1.38E-02	32.43
9-KSTGGKAPR-17	514.2982	514.2984	2	-0.25	K9(me2), K14(ac)	Ss_H3.1, Ss_H3.3	5.21E-02	22.37
9-KSTGGKAPR-17	521.3059	521.3062	2	-0.61	K9(me3), K14(ac)	Ss_H3.1, Ss_H3.3	1.88E-01	22.19
9-KSTGGKAPR-17	528.3127	528.3140	2	-2.49	K9(me2), K14(me1)	Ss_H3.1, Ss_H3.3	1.11E-01	23.63
9-KSTGGKAPR-17	556.3089	556.3089	2	-0.02	K9(me1), S10(ac), K14(ac)	Ss_H3.1, Ss_H3.3	5.12E-02	34.32
9-KSTGGKAPR-17	563.3163	563.3167	2	-0.72	K9(me1), S10(ac)	Ss_H3.1, Ss_H3.3	4.05E-02	35.72
9-KSTGGKAPR-17	563.3163	563.3167	2	-0.72	S10(ac), K14(me1)	Ss_H3.1, Ss_H3.3	4.05E-02	35.89
9-KSTGGKAPR-17	549.3190	549.3193	2	-0.47	K9(me3), S10(ac)	Ss_H3.1, Ss_H3.3	9.75E-02	28.73
18-KQLATKAAR-26	570.8407	570.8404	2	0.49	K18(ac)	Ss_H3.1, Ss_H3.3	5.41E-04	39.76
18-KQLATKAAR-26	584.8559	584.8560	2	-0.28	K18(me1)	Ss_H3.1, Ss_H3.3	1.67E-02	43.96
18-KQLATKAAR-26	598.8534	598.8535	2	-0.09	T22(ac)	Ss_H3.1, Ss_H3.3	1.86E-02	43.74
18-KQLATKAAR-26	570.8407	570.8404	2	0.49	K23(ac)	Ss_H3.1, Ss_H3.3	5.41E-04	40.00
18-KQLATKAAR-26	584.8558	584.8560	2	-0.41	K23(me1)	Ss_H3.1, Ss_H3.3	5.87E-03	43.49
18-KQLATKAAR-26	570.8576	570.8586	2	-1.62	K23(me3)	Ss_H3.1, Ss_H3.3	1.56E-01	36.06
18-KQLATKAAR-26	563.8325	563.8326	2	-0.09	K18(ac), K23(ac)	Ss_H3.1, Ss_H3.3	2.35E-04	38.51
18-KQLATKAAR-26	605.8616	605.8613	2	0.50	T22(ac), K23(me1)	Ss_H3.1, Ss_H3.3	1.36E-03	45.43
18-KQLATKAAR-26	598.8535	598.8535	2	0.02	K18(ac), T22(ac), K23(me1)	Ss_H3.1, Ss_H3.3	7.01E-03	43.61
27-KSAPATGGVKKPHR-40	836.4815	836.4806	2	1.03	K27(me1)	Ss_H3.1	1.72E-03	36.22
27-KSAPATGGVKKPHR-40	543.9862	543.9860	3	0.33	K27(me2)	Ss_H3.1	4.82E-03	29.32

27-KSAPATGGVKKPHR-40	548.6573	548.6579	3	-1.05	K27(me3)	Ss_H3.1	1.29E-02	29.23
27-KSAPATGGVKKPHR-40	836.4815	836.4806	2	1.03	K36(me1)	Ss_H3.1	1.72E-03	36.10
27-KSAPATGGVKKPHR-40	548.6577	548.6579	3	-0.37	K36(me3)	Ss_H3.1	7.09E-02	30.66
27-KSAPATGGVKKPHR-40	829.4728	829.4728	2	-0.05	K27(me1), K36(ac)	Ss_H3.1	1.63E-02	35.21
27-KSAPATGGVKKPHR-40	539.3140	539.3141	3	-0.17	K27(me2), K36(ac)	Ss_H3.1	6.46E-02	28.59
27-KSAPATGGVKKPHR-40	843.4887	843.4885	2	0.25	K27(me1), K36(me1)	Ss_H3.1	2.92E-03	37.44
27-KSAPATGGVKKPHR-40	548.6577	548.6579	3	-0.37	K27(me2), K36(me1)	Ss_H3.1	7.09E-02	30.52
27-KSAPATGGVKKPHR-40	553.3293	553.3298	3	-0.78	K27(me3), K36(me1)	Ss_H3.1	5.28E-02	30.89
27-KSAPATGGVKKPHR-40	562.6611	562.6614	3	-0.52	K27(me3), S28(ac)	Ss_H3.1	3.16E-02	32.47
27-KSAPTTGGVKKPHR-40	837.4700	837.4703	2	-0.26	K27(ac)	Ss_H3.3	3.58E-03	33.14
27-KSAPTTGGVKKPHR-40	851.4844	851.4859	2	-1.73	K27(me1)	Ss_H3.3	4.64E-03	36.18
27-KSAPTTGGVKKPHR-40	553.9889	553.9895	3	-1.12	K27(me2)	Ss_H3.3	2.65E-02	29.55
27-KSAPTTGGVKKPHR-40	558.6602	558.6614	3	-2.13	K27(me3)	Ss_H3.3	1.78E-02	29.37
27-KSAPTTGGVKKPHR-40	837.4700	837.4703	2	-0.26	K36(ac)	Ss_H3.3	3.58E-03	33.32
27-KSAPTTGGVKKPHR-40	851.4857	851.4859	2	-0.23	K36(me1)	Ss_H3.3	3.03E-03	35.59
27-KSAPTTGGVKKPHR-40	558.6606	558.6614	3	-1.41	K36(me3)	Ss_H3.3	2.13E-01	30.70
27-KSAPTTGGVKKPHR-40	844.4778	844.4781	2	-0.34	K27(me1), K36(ac)	Ss_H3.3	1.16E-02	35.21
27-KSAPTTGGVKKPHR-40	858.4937	858.4937	2	-0.07	K27(me1), K36(me1)	Ss_H3.3	1.44E-03	37.36
27-KSAPTTGGVKKPHR-40	558.6606	558.6614	3	-1.41	K27(me2), K36(me1)	Ss_H3.3	2.13E-01	30.57
27-KSAPTTGGVKKPHR-40	563.3321	563.3333	3	-2.14	K27(me3), K36(me1)	Ss_H3.3	1.49E-01	30.70
27-KSAPTTGGVKKPHR-40	872.4910	872.4912	2	-0.24	K27(me1), S28(ac)	Ss_H3.3	2.97E-02	37.44
27-KSAPTTGGVKKPHR-40	572.6641	572.6649	3	-1.45	K27(me3), S28(ac)	Ss_H3.3	3.19E-02	32.56
27-KSAPTTGGVKKPHR-40	577.3364	577.3368	3	-0.69	K27(me3), S28(ac), K36(me1)	Ss_H3.3	2.93E-01	33.94
41-YRPGTVALR-49	572.8275	572.8273	2	0.33	Y41(ac), R42(me1)	Ss_H3.3	4.79E-02	42.38
53-KYQKSTELLIR-63	801.9598	801.9587	2	1.37	K53(me1), Y54(ac)	Ss_H3.1, Ss_H3.3	9.05E-04	49.79
53-KYQKSTELLIR-63	801.9598	801.9587	2	1.37	Y54(ac), K56(me1)	Ss_H3.1, Ss_H3.3	9.05E-04	49.89
