# Supplementary material for: Analysis of Histones H3 and H4 Reveals Novel and Conserved Post-Translational Modifications in Sugarcane
Source: PLoS One. 2015 Jul 30;10(7):e0134586. doi: 10.1371/journal.pone.0134586 (PMC4520453; doi:10.1371/journal.pone.0134586)
Supplement: S4 Table — (PDF) [file pone.0134586.s011.pdf]

S4 Table. List of modified peptides corresponding to sugarcane histone H4 identified in the nanoLC-MS/MS analysis of bulk histones.

Sequence	Measured *m/z*	Calculated *m/z*	Charge	ppm	Modification	Histone type	Score	Retention time (min)
4-GKGGKGLGKGGAKR-17	768.9466	768.9464	2	0.28	K5(ac)	Ss_H4.1	1.06E-03	36.90
4-GKGGKGLGKGGAKR-17	768.9646	768.9646	2	0.01	K5(me3)	Ss_H4.1	7.93E-02	34.03
4-GKGGKGLGKGGAKR-17	768.9466	768.9464	2	0.28	K8(ac)	Ss_H4.1	1.06E-03	36.94
4-GKGGKGLGKGGAKR-17	768.9646	768.9646	2	0.01	K8(me3)	Ss_H4.1	7.93E-02	34.03
4-GKGGKGLGKGGAKR-17	768.9466	768.9464	2	0.28	K12(ac)	Ss_H4.1	1.06E-03	37.31
4-GKGGKGLGKGGAKR-17	768.9646	768.9646	2	0.01	K12(me3)	Ss_H4.1	7.93E-02	34.03
4-GKGGKGLGKGGAKR-17	768.9466	768.9464	2	0.28	K16(ac)	Ss_H4.1	1.06E-03	37.19
4-GKGGKGLGKGGAKR-17	768.9646	768.9646	2	0.01	K16(me3)	Ss_H4.1	7.93E-02	34.03
4-GKGGKGLGKGGAKR-17	761.9385	761.9386	2	-0.16	K5(ac), K8(ac)	Ss_H4.1	2.64E-02	35.85
4-GKGGKGLGKGGAKR-17	761.9385	761.9386	2	-0.16	K5(ac), K12(ac)	Ss_H4.1	2.64E-02	35.81
4-GKGGKGLGKGGAKR-17	761.9385	761.9386	2	-0.16	K5(ac), K16(ac)	Ss_H4.1	2.64E-02	36.06
4-GKGGKGLGKGGAKR-17	761.9385	761.9386	2	-0.16	K8(ac), K12(ac)	Ss_H4.1	2.64E-02	35.72
4-GKGGKGLGKGGAKR-17	761.9385	761.9386	2	-0.16	K8(ac), K16(ac)	Ss_H4.1	2.64E-02	35.94
4-GKGGKGLGKGGAKR-17	761.9385	761.9386	2	-0.16	K12(ac), K16(ac)	Ss_H4.1	2.64E-02	35.94
4-GKGGKGLGKGGAKR-17	754.9307	754.9308	2	-0.15	K8(ac), K12(ac), K16(ac)	Ss_H4.1	8.99E-03	34.69
4-GKGGKGLGKGGAKR-17	754.9307	754.9308	2	-0.15	K5(ac), K12(ac), K16(ac)	Ss_H4.1	8.99E-03	34.65
4-GKGGKGLGKGGAKR-17	754.9307	754.9308	2	-0.15	K5(ac), K8(ac), K16(ac)	Ss_H4.1	8.99E-03	34.73
4-GKGGKGLGKGGAKR-17	754.9307	754.9308	2	-0.15	K5(ac), K8(ac), K12(ac)	Ss_H4.1	8.99E-03	34.65
4-GKGGKGLGKGGAKR-17	747.9230	747.9230	2	0.10	K5(ac), K8(ac), K12(ac), K16(ac)	Ss_H4.1	3.57E-03	33.36
18-HRKVLR-23	302.8591	302.8592	3	-0.37	K20(ac)	Ss_H4.1	2.05E-01	24.02
20-KVLRDNIQGITKPAIR-35	988.5863	988.5862	2	0.13	K20(ac)	Ss_H4.1	5.05E-03	43.78
20-KVLRDNIQGITKPAIR-35	659.4053	659.4016	3	-5.75	K20(me3)	Ss_H4.1	3.87E-15	39.54
24-DNIQGITKPAIR-35	747.4198	747.4197	2	0.05	T30(ac)K31(me1)	Ss_H4.1	3.65E-03	45.93
